# Proteasome activator PA200 maintains stability of histone marks during transcription and aging

**DOI:** 10.7150/thno.48744

**Published:** 2021-01-01

**Authors:** Tian-Xia Jiang, Shuang Ma, Xia Han, Zi-Yu Luo, Qian-Qian Zhu, Tomoki Chiba, Wei Xie, Kui Lin, Xiao-Bo Qiu

**Affiliations:** 1 State Key Laboratory of Cognitive Neuroscience & Learning and Ministry of Education Key Laboratory of Cell Proliferation & Regulation Biology, College of Life Sciences, Beijing Normal University, 19 Xinjiekouwai Avenue, Beijing 100875, China.; 2 College of Life Sciences, Beijing Normal University, 19 Xinjiekouwai Avenue, Beijing 100875, China.; 3 Graduate School of Life and Environmental Sciences, University of Tsukuba, 1-1-1 Tennodai, Tsukuba, Ibaraki 305-8577, Japan.; 4 School of Life Sciences, Tsinghua University, Beijing 100084, China.

**Keywords:** Histone marks, aging, histone degradation, proteasome activator PA200, transcription

## Abstract

The epigenetic inheritance relies on stability of histone marks, but various diseases, including aging-related disorders, are usually associated with alterations of histone marks. Whether and how the proteasome is responsible for maintaining the histone marks during transcription and aging remain unclear. The core histones can be degraded by the atypical proteasome, which contains the proteasome activator PA200, in an acetylation-dependent manner during somatic DNA damage response and spermiogenesis.

**Methods:** By utilizing a substitute of methionine to label proteins metabolically, we analyzed histone degradation genome-wide by sequencing the DNA fragments following pulse-chase assays. The genome-wide RNA-sequencing analysis was performed to analyze transcription and chromatin-immunoprecipitation (ChIP)-sequencing was used for analyses of histone marks. The experimental models included gene-manipulated cells (including both mouse and yeast), mouse liver, and mice.

**Results:** Degradation of H4 or the transcription-coupled histone variant H3.3 could be suppressed by deletion of PA200 or its yeast ortholog Blm10. The histone deacetylase inhibitor accelerated the degradation rates of H3, while the mutations of the putative acetyl-lysine-binding region of PA200 abolished histone degradation in the G1-arrested cells. Deletion of PA200 dramatically altered deposition of the active transcriptional hallmarks (H3K4me3 and H3K56ac) and transcription, especially during cellular aging. Furthermore, deletion of PA200 or Blm10 accelerated cellular aging. Notably, the PA200-deficient mice displayed a range of aging-related deteriorations, including immune malfunction, anxiety-like behavior and shorter lifespan.

**Conclusion:** PA200 promotes the transcription-coupled degradation of the core histones, and plays an important role in maintaining the stability of histone marks during transcription and aging.

## Introduction

The current model for epigenetic inheritance relies on stability of histone marks. The core histones, including H2A, H2B, H3 and H4, form an octamer to pack DNA into the nucleosome, the basic unit of chromatin 1. Post-translational modifications of histones (i.e., histone marks) regulate various cellular processes, including epigenetic regulation of transcription. For example, H3K4me3 and H3K56ac mark transcription initiation 2 and transcriptionally-active chromatin areas 3, respectively. Histone marks remain relatively stable in a specific type of cell or tissue, and should only be rearranged during development or cellular reprogramming 4, 5. However, aging and various diseases are usually associated with alterations of histone marks 6, 7. Replacement of histone variants often occurs during transcription. Histone variant H3.3, which is expressed and incorporated into the chromatin in a replication-independent manner, is associated with transcriptional activation in higher eukaryotes 8. The transcription-coupled replacement of H3.1 with H3.3 occurs at gene bodies, promoters, and enhancers 9. H3.3 incorporation into nucleosomes marks the transcriptionally-active chromatin to control neuronal synaptic connectivity and cognition 10. It is a mystery how the stability of histone marks is maintained.

Proteasomes catalyze degradation of most cellular proteins, and consist of a 20S catalytic particle and one or two activators, such as the 19S regulatory particle, PA28α/β, PA28γ and PA200/PSME4 11, 12. The typical 26S proteasome with the 19S particle as the activator promotes the ubiquitin-dependent protein degradation 13. PA200 and its yeast ortholog, Blm10, bind to the ends of the 20S particle 14, 15. PA200 accumulates on chromatin in response to DNA damage 16. We have recently shown that the core histones can be degraded by the PA200-proteasome in an acetylation-dependent manner during somatic DNA damage response and spermiogenesis 17. During elongation of spermatids, most core histones are degraded by the specialized PA200-containing proteasomes (i.e., spermatoproteasomes) 17. The acetylation-dependent histone degradation can also occur during DNA damage-induced replication stress 18. In response to DNA damage, the levels of histones from chromatin drop 20-40% in a manner depending on the INO80 nucleosome remodeler 19. In addition, histones are partially lost across the genome during aging in both yeast and human cells 20, 21. It remains unclear how histone loss or degradation influences the stability of histone marks.

In this study, we show that the core histones are degraded in the G1-arrested cells, and present evidence that PA200 promotes the transcription-coupled degradation of the core histones, and maintains the stability of histone marks during transcription and aging.

## Methods

Detailed methods are available in Supplementary Materials.

### Strains and Cell Culture

MEF cells were cultured in Dulbecco's modified Eagle's medium (DMEM) supplemented with 10% fetal bovine serum (FBS), 100 U/ml penicillin, 100 μg/ml streptomycin, 1% non-essential amino acids and 200 μM of β-mercaptoethanol. Primary PA200^+/+^, PA200^-/-^ MEF cells, and PA28γ^-/-^ MEF cells were isolated from the embryos of the wild-type, PA200-deficient mice and PA28γ-deficient mice, respectively. The permanent wild-type and PA200-deficient MEF cells were obtained from the mice as described 17. The PA28γ-deficient cells were obtained from Drs. Lance Barton and Xiaotao Li. The ATG5-deficient MEF cells were obtained from Dr. Alfred L. Goldberg, who received the cells from Dr. Yoshinori Ohsumi.

All yeast strains were grown at 30°C in yeast peptone dextrose (YPD). The yeast strain BY4741 (MATa his3△1 leu2△0 met15△0 ura3△0) was from Dr. Wei Li. The strain YHS539 (MATa BY4741 yak1-GFP (kan), blm10::Nat hat9) was obtained from Dr. Daniel Finley. The yeast strain Blm10 O/E (MATa BY 4741 blm10::NAT GPD-HA-Blm10) was obtained as described 17.

### Pulse-chase assay

The pulse-chase assay was employed to determine histone degradation by labeling proteins with Aha 22. Briefly, the G1-arested MEF cells cultured in MEF medium with minosine (0.2 μM, Sigma-Aldrich, #M0253) were first subjected to starvation with the media containing all amino acid except 0.2 mM methionine (Met) for 30 min. Then, the medium was replaced with MEF medium containing Aha (0.2 mM, Anaspec, #63669), instead of Met, for 2 h. After Aha treatment, the MEF medium with 0.2 mM Met was used to replace the medium with Aha. Because macroautophagy can remove the cytosolic fraction of the core histones 23, we used acid extraction, which could efficiently extract the core histones from the chromatin fraction, to exclude the cytosolic fraction of the core histones 24. The extracted core histones in this step are the total histones. Then, we ligated chromatin histones with biotin through the copper-catalyzed cycloaddion reaction. After washing the bead-bound nucleosomes with a solution of 4 M urea and 0.3 M NaCl to remove H2A/H2B dimers and other DNA-bound proteins, only H3/H4 tetramers and associated DNAs remained. The Aha-containing histones of chromatin were captured by streptavidin-coated beads, and were finally incubated with 1× SDS buffer at 95 ºC for 5 min. The samples were analyzed by immunoblotting to detect the corresponding proteins. We analyzed H3.3 and H3 from streptavidin-coated beads by using their corresponding antibodies. The input H3.3 and H3 were set as controls. The degradation of histones was additionally monitored by using the anti-streptavidin antibody. To show the equal loading, the input biotin-labeled histones were analyzed by Coomassie blue staining.

### Genome-wide analysis of histone degradation (GAHD)

After pulse-chase analysis as described as above, the DNA fragments associated with the newly-synthesized H3/H4 proteins were then purified with DNA purification columns (CST, #14209) and processed for ChIP-seq as described later.

We used MAnorm to statistically compare the signal density of ChIP-seq data at chase times 0 and 4 h. To identify the degraded region of the G1-arrested cells, we selected the unique peaks which were present in ChIP-seq at 0 h, but were absent at 4 h. We selected the common peaks whose read density at 0 h was significantly larger than that at 4 h. Then, we defined the PA200-depdentdent histone degradation regions, which were not overlapped between the wild-type and the PA200^-/-^ samples.

### Yeast replicative life span analysis

Old yeast cells were isolated by using EZ Link Sulfo-NHL-LC-LC-Biotin (Thermo Scientific) to label cell surface proteins (Invitrogen) 25. The older cells were isolated by several rounds of this affinity purification. Ages of isolated cells were estimated by counting Calcofluor-stained bud scars as described previously 20. Proteins on the surface of logarithmic phase cells were labeled with EZ Link Sulfo-NHL-LC-LC-Biotin. The old mother cells were isolated by Dynabeads Streptavidin T1, and stained with calcofluor 28 (Sigma) for counting the bud scar numbers.

### Immunoblotting assay

Cells were lysed in the buffer containing 20 mM Tris-HCl, pH 8.0, 100 mM KCl, 0.2% Nonidet P-40, 10% glycerol, 1 mM ZnCl_2_, 10 mM β-glycerophosphate, 5 mM tetrasodium pyrophosphate, 1 mM NaF, 1 mM Na_3_VO_4_, and a mixture of protease inhibitors. Protein samples were separated by SDS-PAGE and transferred onto PVDF membranes. Antibodies or antisera against specific proteins were used as primary antibodies to detect the corresponding proteins. Peroxidase-conjugated anti-mouse IgG (1:5000) or anti-rabbit IgG (1:5000) was used as the secondary antibody. The protein bands were visualized by ECL detection system (Millipore) or ODYSSEY (LI-COR).

### Immunofluorescence analysis

Cells were plated on coverslips, cultured in a six-well plate and then subjected to appropriate treatments. Cells were fixed with 4% formaldehyde, blocked with 5% normal goat serum for 60 min at 37 °C, and then incubated with anti-PA200 or anti-H4K16ac antibody overnight at 4 °C, followed by incubation with Alexa fluor 594-conjugated anti-rabbit IgG or Alexa fluor 488-conjugated anti-mouse IgG for 60 min at room temperature. The nucleus was stained with DAPI. Finally, the coverslips were examined with a confocal laser scanning microscope.

### Data availability

All sequencing data, including RNA-seq, ChIP-seq, genome-wide analysis of histone degradation, and WGBS, have been deposited at the BioProject database (https://www.ncbi.nlm.nih.gov/bioproject) with accession number PRJNA451247. The data that support the findings of this study are available from the authors upon request. Correspondence and requests for materials should be addressed to X.B.Q. (xqiu@bnu.edu.cn) or K.L. (linkui@bnu.edu.cn).

## Results

### PA200-proteasome promotes acetylation-dependent degradation of core histones in G1-arrested cells

**Figure 1:**
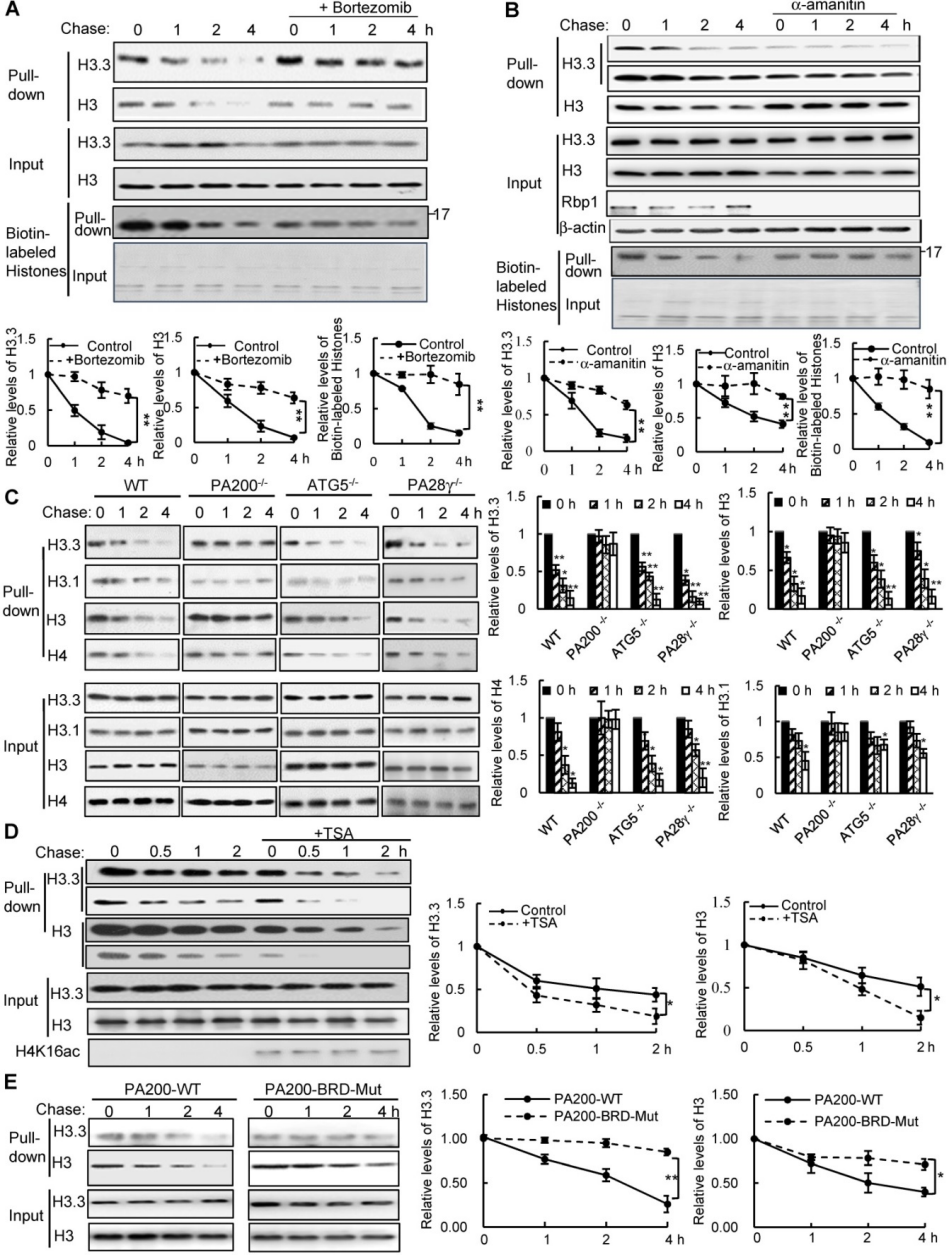
** PA200 promotes acetylation-dependent degradation of core histones during transcription. (A)** The G1-arrested MEF cells were pulse-labeled with Aha and then chased in the Met-containing medium in the absence or presence of 0.1 µM of Bortezomib for the time indicated. Bortezomib was added for 4 h at all-time points of chases. Histones were captured by streptavidin-coupled beads and analyzed by immunoblotting. The corresponding input histone were used as the loading control. The captured histones levels were quantified by densitometry (normalized to the corresponding input histones). ** *p<*0.01; two-way ANOVA. **(B)** Histone degradation in the G1-arrested MEFs treated with the transcription inhibitor α-amanitin at the concentration of 10 mM for 24 h was analyzed by the pulse-chase assay**.** Histones captured by streptavidin-coupled beads (*i.e.*, pull-down) were analyzed by immunoblotting with corresponding antibodies. The input biotin-labeled histones were analyzed by Coomassie blue staining to show the equal loading (A-B). The captured histones levels were quantified by densitometry (normalized to the corresponding input histone). ** *p<*0.01; two-way ANOVA. **(C)** Histone degradation in the G1-arrested wild-type, PA200^-/-^, ATG5^-/-^, and PA28γ^-/-^ MEF cells was analyzed by the pulse-chase assay. Protein levels were analyzed by immunoblotting as in (A). * *p<*0.05, ** *p<*0.01; one-way ANOVA. **(D)** The G1-arrested MEF cells were incubated in the absence or presence of 0.3 μM of TSA for 4 h. Histone degradation was analyzed by the pulse-chase assay. Protein levels were quantified by densitometry (normalized to input histones). * *p<*0.05; two-way ANOVA. **(E)** Histone degradation in the G1-arrested 293T transfected with either the wild-type or mutant PA200 (PA200-BRD-Mut) with a HA-tag was analyzed by the pulse-chase assay. Protein levels were analyzed by immunoblotting as in (A). * *p<*0.05, ** *p<*0.01; two-way ANOVA. The relative histone levels in different groups at time point 0 were set to 1. All biotin-labeled histones in pull-down experiments were probed by the anti-streptavidin antibody, and the input biotin-labeled histones were analyzed by Coomassie blue staining in (A-B). Data represent three independent biological replicates (mean ± SEM).

To test whether histones are degraded during transcription, we took advantage of a modified pulse-chase assay by metabolically labeling proteins with a substitute of methionine (Met), azidohomoalanine (Aha). In this assay, Aha was co-translationally incorporated into proteins and subsequently ligated with biotin. Thus, following chase in the regular medium with Met, old histones with Aha could be purified with streptavidin for analysis of degradation (Figure S1A). In the G1-arrested mouse embryonic fibroblast (MEF) cells, the levels of histone H3 decreased during chase (Figure 1A-C and Figure S1B). Histone variant H3.3, which can be incorporated into chromatin independently of replication, was also degraded in the G1-arrested MEF cells (Figure 1A-C). Treatment with the proteasome inhibitor, Bortezomib (also named Velcade), blocked their degradation (Figure 1A), suggesting that the proteasome mediates histone degradation in the G1-arrested MEF cells. Addition of Bortezomib increased the levels of histones at 0 h probably by blocking histone degradation, but decreased the levels of biotinylated histones probably by reducing the integration of Aha into proteins. Furthermore, the transcription inhibitor, α-amanitin, which induces degradation of the largest RNA polymerase II subunit Rbp1 26, suppressed degradation of histones H3.3 and H3 (Figure 1B).

Because amanitin was added for 24 h before pulse-chase analysis, Rbp1 was not detectable even at 0 h of the chase. Probably because H3.3, but not other species of H3 (*e.g*., H3.1), is only integrated into chromatin during transcription 8, the levels of H3.3, but not of H3 in general, in the amanitin-treated cells decreased markedly at 0 h of the chase, supporting the notion that H3.3 is integrated into the chromatin during transcription much more than other H3 species. However, other H3 species are still present in the chromatin and are still degradable, even at a much slower rate. Thus, the degradation of both H3.3 and H3 was suppressed in amanitin-treated samples following a chase (Figure 1B). These results strongly support that the degradation of the core histones depends on transcription.

We previously showed that the PA200-proteasomes promote the acetylation-dependent degradation of the core histones during DNA repair and spermiogenesis 17. Deletion of PA200 suppressed the degradation of H3.3 and histone H4 in the G1-arrested cells (Figure 1C). The canonical histone H3 variant H3.1, which is incorporated during replication 27, showed a slight degradation. This degradation was also suppressed by PA200 deletion (Figure 1C and Figure S1C). However, deletion of another proteasomal activator, PA28γ, showed no inhibitory effects on histone degradation (Figure 1C and Figure S1C), supporting the specific role of PA200 in histone degradation in the G1-arrested MEF cells. Lamin B1 is a nuclear protein, which undergoes the autophagy-mediated degradation 28. We found that the degradation of lamin B1 could not be blocked by deletion of PA200 or PA28γ (Figure S1D). A small fraction of histones is present in the cytosol and can be removed by macroautophagy, which requires the autophagic gene ATG5 23. Our method using acid extraction could efficiently extract the core histones from the chromatin fraction to exclude the influence of the cytosolic fraction 24. Deletion of ATG5 had no effect on the degradation of chromatin histones, but suppressed degradation of lamin B1 (Figure 1C and Figure S1D). Furthermore, trichostatin A (TSA), a histone deacetylase inhibitor, accelerated the degradation rates of H3 in general, especially of its variant H3.3 (Figure 1D and Figure S1E). PA200 bears an acetyl-lysine-binding bromodomain-like (BRDL) region with critical residues at N1716 and F1717 17. The mutations at this BRDL region (N1716T/F1717S) of PA200 abolished histone degradation in the G1-arrested cells (Figure 1E and Figure S1F). The BRDL mutant had a more prominent effect on transcription-specific H3.3 than H3. We showed previously that PA200 directly associates with the acetylated core histones in the unsynchronized cells 17. Similarly, PA200 also colocalized with the acetylated histone H4K16ac during transcription in the G1-arrested cells (Figure S1G*).* These results further support our previous notion that acetylation is involved in the degradation of the core histones by the PA200-proteasome 17.

### PA200 promotes degradation of core histones primarily in actively-transcribed regions

**Figure 2:**
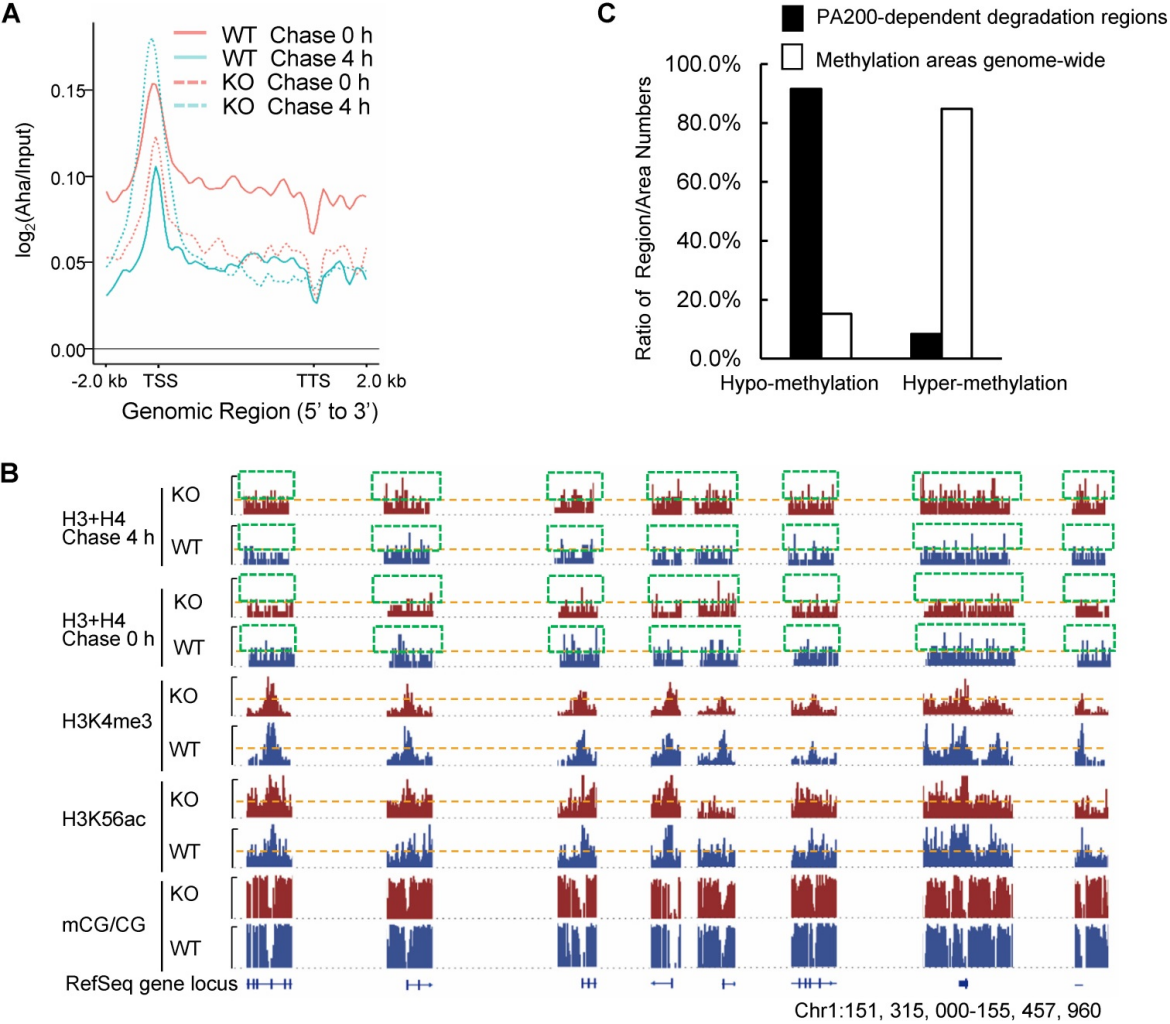
** PA200 promotes degradation of core histones primarily in actively-transcribed regions. (A)** Gene plot of core histone enrichment signals in PA200^+/+^ (WT) and PA200^-/-^ (KO) MEFs.** (B)** The IGV genome browser view of H3K4me3 and H3K56ac enrichment in WT and PA200 KO MEFs for the selected chromosomes, while other regions between them are hidden as indicated by grey dash lines. The levels of DNA methylation are shown in parallel. Yellow dash lines indicate equal heights at y axes in each pair of WT and PA200 KO samples. Squares with green dash lines highlighted the differences in the levels of histones H3 and H4 in the selected clusters of gene loci. The signal track of each sample was calculated by MACS2 and normalized by sequencing depth. **(C)** The distribution of PA200-dependent histone degradation regions genome-wide by collectively analyzing DNA sequencing and WGBS data. The PA200-dependent histone degradation regions include 5972 hypo-methylation regions and 550 hyper-methylation regions, which are defined in the Methods. The average CG levels within the “hyper-methylation” regions are >0.5. To analyze the distribution of hypo-/hyper-methylation areas genome-wide, the areas whose average CG levels are>0.5 are defined as “hyper-methylation areas”, whereas whose average CG levels are<0.5 are defined as “hypo-methylation areas” as described in the Methods. The numbers of hyper-methylation and hypo-methylation areas are 684 and 123, respectively.

By utilizing Aha to label proteins metabolically, we performed genome-wide analysis of histone degradation (GAHD) by sequencing the DNA fragments purified together with histones following 2 h-pulse labeling with Aha and chase in the regular medium for 0 or 4 h. In the wild-type MEF cells at 0-h chase, the levels of the Aha-labeled core histones (primarily H3 and H4) peaked at transcriptional start sites (TSS) genome-wide (Figure 2A). In the wild-type MEF cells at 4-h chase, the levels of the Aha-labeled core histones dramatically dropped, but maintained at a similar pattern in coding regions to that at 0-h chase (Figure 2A). In the PA200-deficient MEF cells at 0-h chase, the enrichment of the Aha-labeled core histones was much lower throughout the coding regions than that in the wild-type cells. In the PA200-deficient MEF cells at 4-h chase, the levels of the Aha-labeled core histones just slightly dropped in coding regions, supporting the notion that PA200 promotes degradation of the core histones during transcription. Surprisingly, the levels of the Aha-labeled core histones were raised at TSS (transcriptional start site) in the PA200-deficient MEF cells after 4-h chase (Figure 2A). Given that the nucleosome turnover by which the core histones are often disassembled from coding regions and then reassembled during transcription is exceptionally active at TSS 29, this rise is probably because deletion of PA200 led to accumulation of the Aha-labeled core histones at TSS regions in the process of nucleosome turnover. These results raise the possibility that the PA200-mediated degradation of the core histones facilitates nucleosome turnover.

The IGV genome browser view of these sequencing data further supported the above conclusions. Deletion of PA200 indeed reduced the enrichment of the Aha-labeled core histones in many genome regions, as evidenced by the reduced levels of the Aha-labeled core histones (e.g., areas highlighted by green squares) in the PA200-deficinet MEF cells at 0 h of chase (Figure 2B). After 4-h chase, deletion of PA200 markedly attenuated the drop in the levels of the Aha-labeled core histones in most genome regions, and increased their levels (e.g., areas highlighted by green squares) at certain genome regions. H3K4me3 and H3K56ac mark transcription initiation 2 and transcriptionally-active chromatin regions 3, respectively. In general, there were only 8% (550/6522 regions) of the PA200-dependent degradation of the core histones was located in the hyper-methylation regions in the G1-arrested cells by analyzing DNA methylation data of whole-genome bisulfite sequencing (WGBS) along with the ChIP-seq data for histones (Figure 2C). The ratio of the hyper-methylation areas genome-wide is 84.76% (684/807 areas) (Figure 2C), suggesting that the core histones are primarily degraded in a PA200-dependent manner in the actively-transcribed regions (i.e., hypo-methylated regions). Taken together, these data demonstrate that the PA200-proteasome promotes the acetylation-dependent degradation of the core histones primarily in the actively-transcribed genome regions, and influences H3K4me3 and H3K56ac enrichment genome-wide.

### PA200 is critical to maintenance of histone marks and regulation of transcription

**Figure 3:**
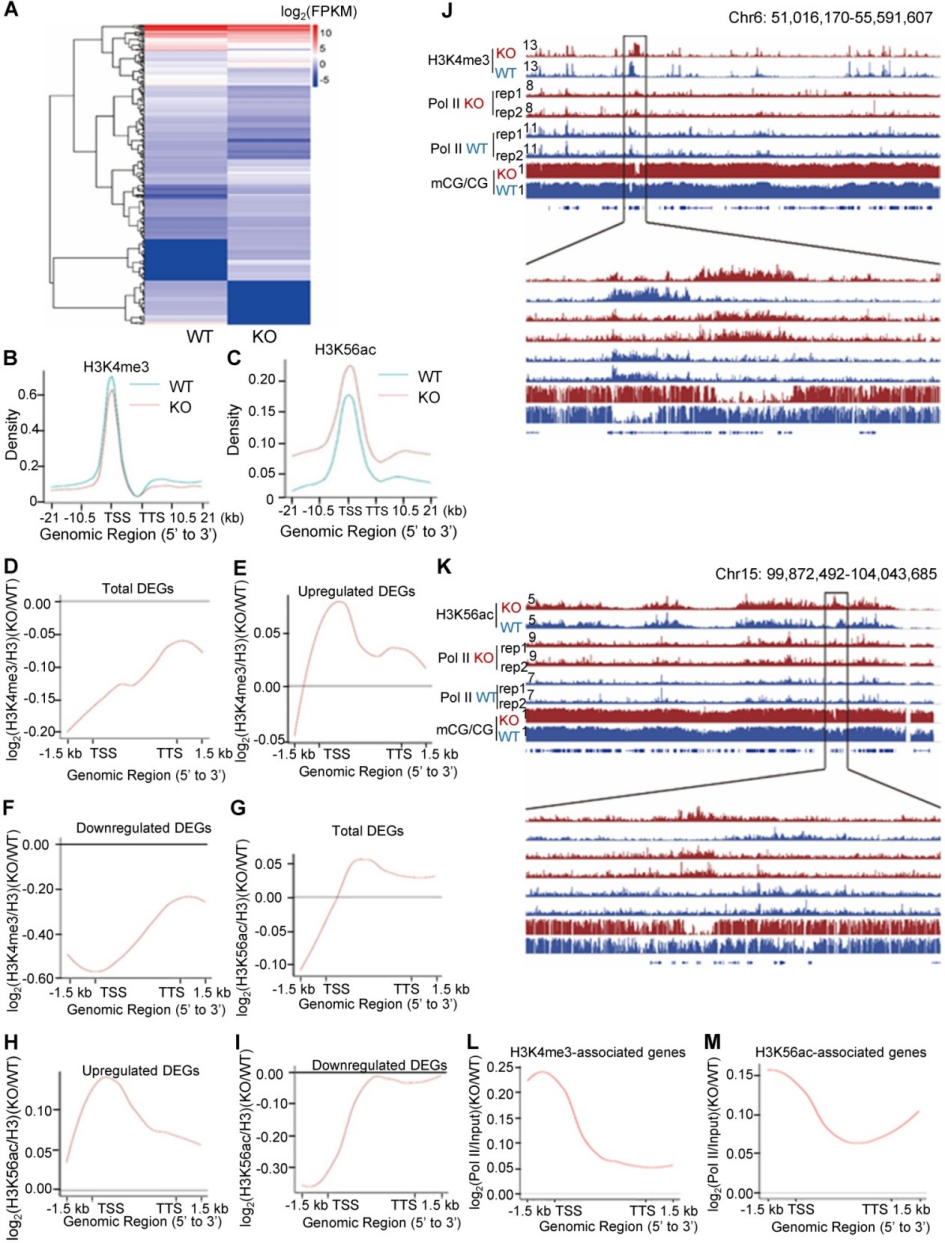
** Deletion of PA200 disrupts genome-wide transcription. (A)** Hierarchical clustering of the differentially-expressed genes (DEGs) in the PA200^+/+^ (WT) and PA200^-/-^ (KO) mouse liver. DEGs were defined according to the combination of the absolute value of log2-Ratio ≥1 and diverge probability ≥0.8. Coloring indicates the log2-transformed fold change. **(B-C)** Enrichment of H3K4me3 (B) and H3K56ac (C) on the gene bodies and 21 kb upstream of TSS and 21kb downstream of TTS regions in the MEF genome. **(D-I)** The genome average plot for the change in H3K4me3 (D-F) or H3K56ac (G-I) (normalized to H3) in the PA200^-/-^ (KO) over the wild-type MEF cells (WT).** (J-K)** The IGV genome browser view of H3K4me3 (J) or H3K56ac (K) enrichment in PA200^+/+^ (WT) and PA200^-/-^ (KO) MEF chromosomes. The levels of DNA methylation and polymerase II were shown in parallel. **(L-M)** The profile analysis of RNA polymerase II on the gene bodies and 1.5 kb upstream of TSS and 1.5 kb downstream of TTS regions of the genes enriched with the H3K4me3 (L) or H3K56ac (M) in the PA200^-/-^ (KO) MEF cells normalized to those in the wild-type group (WT).

To investigate the role of PA200 in regulating transcription, the genome-wide RNA-sequencing analysis was performed using the total RNAs extracted from mouse livers and MEF cells, respectively. Significant changes in the expression of 334 genes were identified in the PA200-deficient livers (Figure 3A and Figure S2A-B). Based on KEGG (Kyoto Encyclopedia of Genes and Genomes) database, functions of these differentially-expressed genes (DEGs) are mainly related to metabolism, transcription, signal transduction, cell growth, and cell death (Figure S2C-D). By analyzing the pathway enrichment of the RNA-seq data, we found that the metabolic pathways involved in the insulin signaling, gluconeogenesis and lipid metabolism were the top enriched pathways in the PA200-deficient livers (Figure S2E). Given that the liver plays critical roles in glucose/lipid metabolism, these results suggest that the PA200-dependent regulation of gene expression is critical to liver function.

Accordingly, deficiency of PA200 in the G1-arrested MEF cells markedly influenced the transcription of 1314 genes (Figure S3A-C). Expression levels of the genes selected from these results, including up-regulated (Tnfrsf1 and Integrin α7) and down-regulated (Rhoj) genes were further validated by quantitative PCR analyses (Figure S3D). These genes regulate important pathophysiological processes, such as aging, metabolism and cancer (Figure S3E-G). Only a few genes were co-up or down-regulated in the PA200-deficient livers and the G1-arrested PA200^-/-^ MEF cells (Figure S3H). Actually, the coverage of the aging pathways is incomplete in these databases. Thus, we searched literatures for the aging-related DEGs of RNA-seq in mouse livers and MEFs, respectively, and the results showed that deletion of PA200 caused the up- or down-regulation of almost completely different sets of the aging-related genes (Table S1). Taken together, the PA200-mediated proteolysis regulates transcription of genes, which dictate many critical pathophysiological activities, at both cell and tissue levels.

To explore the mechanism underlying the above transcriptional changes, we performed chromatin-immunoprecipitation (ChIP)-sequencing analyses of histone marks, which revealed a slight down-regulation of H3K4me3 on the gene bodies and 21 kb upstream of TSS and 21 kb downstream of TTS (transcriptional termination site) regions in the G1-arrested PA200^-/-^ MEF cells (Figure 3B). In contrast, deletion of PA200 led to a marked upregulation of H3K56ac in these genome regions (Figure 3C). Consistently, PA200 deletion markedly elevated protein levels of H3K56ac, but slightly increased the levels of H3K4me3, as revealed by immunoblotting assays (Figure S4A). The up-regulated DEGs were accompanied with the increased H3K4me3 and H3K56ac marks, while the down-regulated DEGs were with the reduced H3K4me3 and H3K56ac marks in the G1-arrested PA200-deficient MEF cells (Figure 3D-I). The promoter levels of the selected up-regulated DEGs (*Hoxa9, Ctnna2, Bmp4*), down-regulated DEGs (*Fzd2, SOD1, Hmgb1*), and not significantly changed genes (*Cers2 and Gpc4*) on H3K4me3 or H3K56ac in the G1-arrested were further validated by quantitative PCR (Figure S4B-C). In contrast, neither the up-regulated DEGs (including *Hoxa9, Ctnna2,* and* Bmp4*) nor the down-regulated DEGs (including *Fzd2, SOD1*, and *Hmgb1*) showed these effects in the PA200-deficient MEF cells that were not arrested (Figure S4B-C). KEGG analysis of the DEGs associated with H3K4me3 or H3K56ac revealed their involvements in the cell cycle, senescence, autophagy, and the cancer- or development-related signal pathways (Figure S5A-D). H3K4me3 and H3K56ac showed similar regulatory tendency on certain up-regulated or down-regulated genes, but displayed distinct tendency on most genes, in response to PA200 deficiency in MEF cells (Figure S5E-F). It is noteworthy that the number for the upregulated genes associated with H3K4me3 or H3K56ac enrichment was 2-3 folds of that for the downregulated genes, hinting an indirect role for PA200 in suppressing expression of most genes because both H3K4me3 and H3K56ac are usually associated with active transcription 30. Notably, changes in the deposition of H3K4me3 or H3K56ac were positively correlated with the recruitment of RNA polymerase II onto chromatins, and inversely correlated with DNA methylation in certain critical gene regions (Figure 3J and 3K, and Figure S6A-H). Representative genes with the coordinated association with both RNA polymerase II and H3K56ac or H3K4me3 (including *Hoxc13* and *Hoxa3*) were further validated by quantitative PCR analysis (Figure S6I-J). Furthermore, the recruitment profile of RNA polymerase II resembled that for the upregulated DEGs in both H3K4me3- and H3K56ac-associated genes in the PA200-deficient MEF cells as shown above (Figure 3E, 3H, 3L, and 3M), suggesting that PA200 regulates transcription by acting at the upstream of RNA polymerase II recruitment.

Deletion of PA200 increased the levels of DNA methylation in general (Figure S7A-C), changed distributions of differential methylation regions (DMRs) (Figure S7D-E), and influenced diverse cellular functions (Figure S7F). Although there were certain overlaps between changes in DMRs and the differentially-expressed genes in PA200^-/-^ MEFs (Figure S7G-H), most hypo-DMR-related genes were upregulated, while a large portion of hyper-DMR-related genes were downregulated (Figure S7G-J). Moreover, the inverse correlations of H3K4me3 or H3K56ac with DMR were primarily observed within gene regions (Figure S7K-O), and a certain portion of this inversion corresponded to the genes that were upregulated (Figure S7P-Q). Although n was 1 in the biological replicates of genome-wide analysis of histone degradation (GAHD), ChIP-sequencing (H3K4me3 or H3K56ac) and WGBS, there were mutual confirmations among the three different assays. For the ChIP-sequencing, there were additionally two biological replicates with the anti-Polymerase II antibody as displayed in Figure 3J, 3K, and S6A-B. Moreover, quantitative PCR analyses have validated these ChIP-sequencing results (Figure S4B, S4C and S6I-J). Taken together, these results suggest that PA200 is critical to the maintenance of histone marks in gene regions and to the regulation of transcription.

### PA200 regulates transcription of aging-related genes

**Figure 4:**
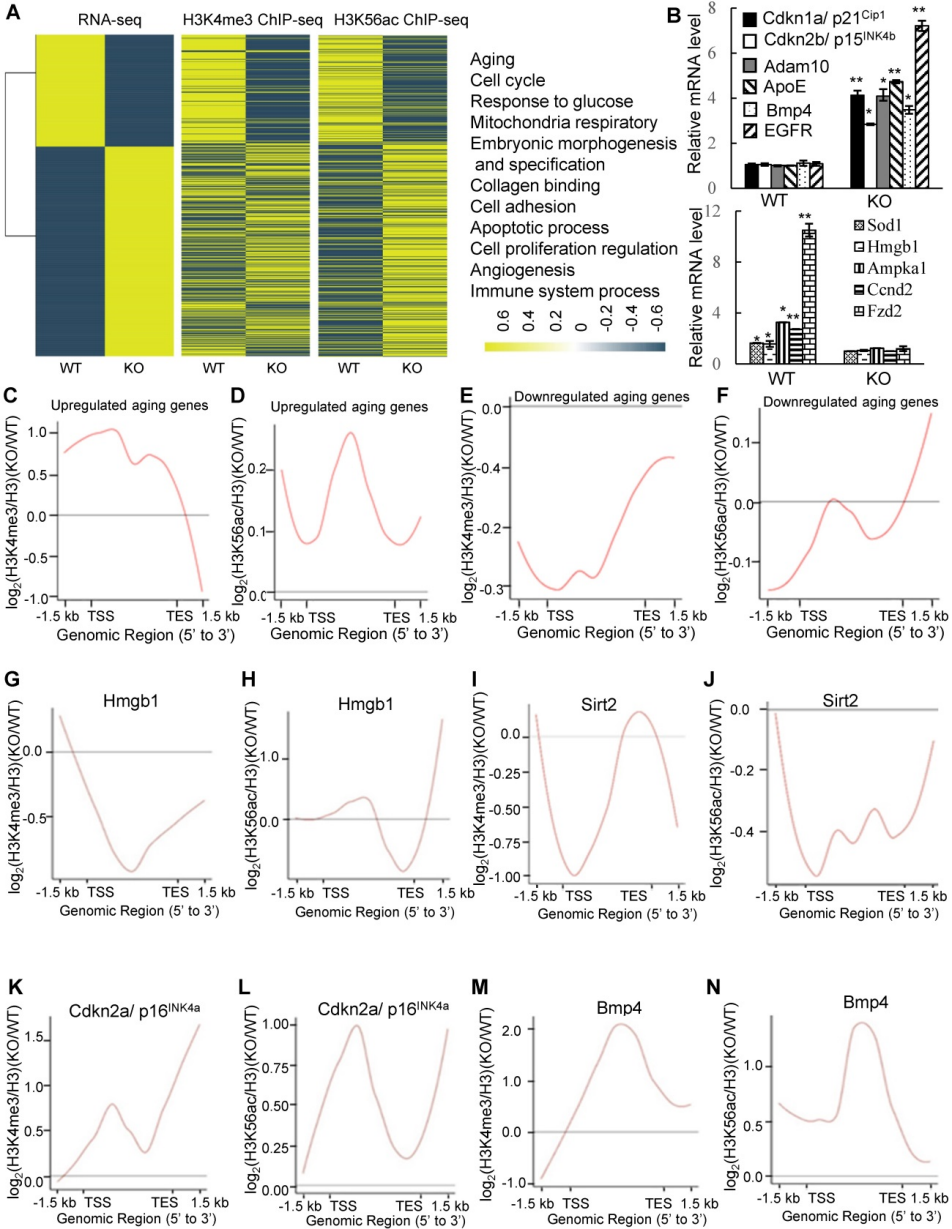
** Deletion of PA200 alters transcription of the aging-related genes. (A)** Heat-map comparison of all DEGs with the H3K4me3 or H3K56ac enrichment at promoters (TSS ± 2.5 kb; middle) in the PA200-deficient MEF cells (normalized to the wild-type group). DEGs were defined according to the combination of the absolute value of log2-ratio ≥1 and diverge probability≥0.8. Coloring indicates the log2-transformed fold change. DEGs were clustered into 11 major groups with enriched GO terms listed (right). All *p* values of GO Terms are <0.0001. **(B)** Quantitative PCR analysis of expression of the aging-related genes in MEF cells. * *p<*0.05, ** *p<*0.01; one-way ANOVA. **(C-F)** The genome average plot for the change in H3K4me3 (C, E) or H3K56ac (D, F) (normalized to H3) on up-regulated or down-regulated aging-related genes in the PA200^-/-^ (KO) normalized to the wild-type group. **(G-N)** The profile analysis of H3K4me3 (G, I, K, M) and H3K56ac (H, J, L, N) on the gene bodies of *hmgb1, sirt2, p16^INK4a^,* and *bmp4* in the PA200^-/-^ (KO) MEF cells normalized to the wild-type group. Data represent three independent biological replicates (mean ± SEM).

To analyze the nature of the genes affected by PA200 deletion, expressions of all genes were grouped into 2 clusters (up- and down-regulated). Enrichment of H3K4me3 or H3K56ac at promoters was generally in a positive correlation with the changes in the RNA levels of specific genes (Figure 4A). Pathway enrichment analysis of DEGs revealed that PA200 deficiency changed the expression of many aging-related genes, such as aging-promoting genes: *Bmp4* 31, *Cdkn1a* (*p21^Cip1^*) 32, *Cdkn2b* (*p15^INK4b^*) 33 and aging-suppressing genes: *Hmgb1* 34 and *Sod1* 35 (Table S1). We further validated these DEGs by quantitative PCR (Figure 4B). The H3K4me3 levels and the H3K56ac levels markedly increased at gene bodies in the up-regulated aging-related genes (Figure 4C-D, and Table S1). The levels of both H3K4me3 and H3K56ac were reduced in the down-regulated aging-related genes in the PA200-deficient MEF cells (Figure 4E-F, and Table S1). Specifically, H3K56ac and H3K4me3 were enriched on the gene bodies of certain aging-promoting genes, such as *Cdkn2a* (*p16^INK4a^*) 36 and *Bmp4*, and decreased on gene bodies of some aging-suppressing genes, such as *Hmgb1* and *Sirt2* 37 in PA200^-/-^ MEF cells (Figure 4G-N). Quantitative PCR analyses confirmed that expression of these aging-related genes was altered accordingly in the PA200-deficient mouse liver (Figure S8A). These results suggest that PA200 regulates the transcription of aging-related genes.

### PA200/Blm10 extends cellular lifespan

**Figure 5:**
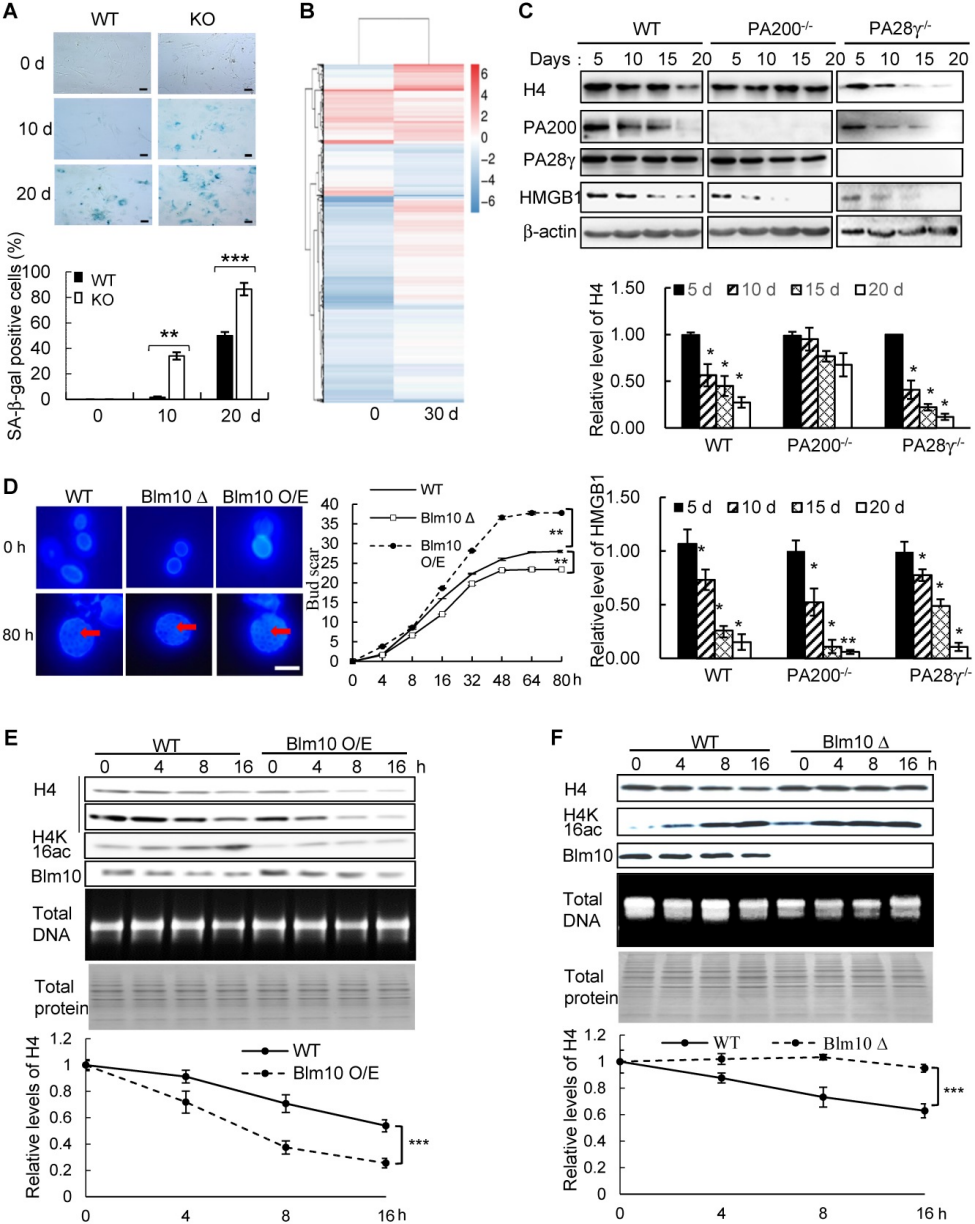
**PA200/Blm10 promotes degradation of the core histones during aging and extends cellular lifespan. (A)** SA-β-gal staining of primary WT and PA200^-/-^ (KO) MEF cells at the indicated days. The percentage of β-galactosidase-positive cells in each group was displayed in bar graph. Scale bar: 50 µm. ** *p<*0.01, *** *p<*0.001; one-way ANOVA. **(B)** Heat map showing the expression (normalized reads per kilo base per million mapped-reads (RPKM)) of all DEGs in PA200^-/-^ (KO) MEF cells cultured for 0 or 30 days (normalized to WT MEFs). Each treatment has two independent biological replicates which were clustered into one group. Coloring indicates the log2 transformed fold change.** (C)** Immunoblotting of the whole-cell extracts from primary MEF cells at indicated days. The levels of HMGB1, a biomarker for young cells, decreased during aging. Protein levels were quantified by densitometry (normalized to β-actin). * *p<*0.05; one-way ANOVA. **(D)** Bud scars of the wild-type, Blm10-deficient (Blm10Δ) or Blm10-overexpressing (Blm10 O/E) yeast at the indicated times. The bar graph showed the bud scar numbers, and images from the calcofluor 28 staining showed the sizes of yeast cells and bud scars. A scar is pointed by an arrow. More than 30 cells were counted for each group. ** *p<*0.01; two-way ANOVA.** (E)** Immunoblotting of the whole-cell extracts of the wild-type or Blm10 O/E at the indicated times. Total sonicated DNA on 1% agarose gel was used as control. The total proteins were visualized by Coomassie staining following SDS-PAGE. Quantitation of histone levels was carried out by densitometry (normalized to total DNA). *** *p<*0.001; two-way ANOVA. **(F)** Immunoblotting of the whole-cell extracts of the wild-type or Blm10Δ yeast as analyzed similarly to (E). Quantitation of histone levels was carried out by densitometry (normalized to total DNA). *** *p<*0.001; two-way ANOVA. The relative histone levels in different groups at time point 0 were set to 1. Data represent three independent biological replicates (mean ± SEM).

We next found that deletion of PA200 reduced the proliferative potential (Figure S8B), and increased the activity of the senescence-associated β-galactosidase (SA-β-gal), a marker for cellular aging 38, in primary MEF cells (Figure 5A). Significant changes in the expression of 3740 genes were identified in the PA200-deficent primary MEFs at days 0 and 30 in comparison to those in the wild-type cells (Figure S8C-D). The expression levels of the selected genes (*SOX9, ICAM-1* and* FXYD6*) in primary MEF cells cultured for 0 (young) or 30 (old) days were further validated by quantitative PCR (Figure S8E). Meanwhile, these changes in DEGs displayed distinct patterns of expression between day 0 and day 30 (Figure 5B). Deletion of PA200 disrupted the expression of the genes related to aging and other pathophysiological processes, including metabolism, development, cancer, and genetic information processing (Figure S8F-H). The levels of H4 usually decrease, but the levels of H4K16ac increase, during aging 20. Notably, deletion of PA200, but not of PA28γ suppressed the loss of H4 during aging (Figure 5C). In order to examine whether this role of PA200 is conserved evolutionally, we showed that deletion of the PA200 ortholog Blm10 decreased, but overexpression of Blm10 increased, the yeast bud scar numbers (Figure 5D), which represent replicative lifespan 20. Overexpression of Blm10 accelerated, but deletion of Blm10 prevented the decrease in the levels of both H4 and H4K16ac during aging (Figure 5E-F and Figure S9), supporting the notion that Blm10 promotes the acetylation-mediated histone degradation during aging. These results suggest that PA200/Blm10 regulates transcription during cellular aging and extends cellular lifespan.

### PA200 deficiency accelerates aging-related pathological changes

**Figure 6:**
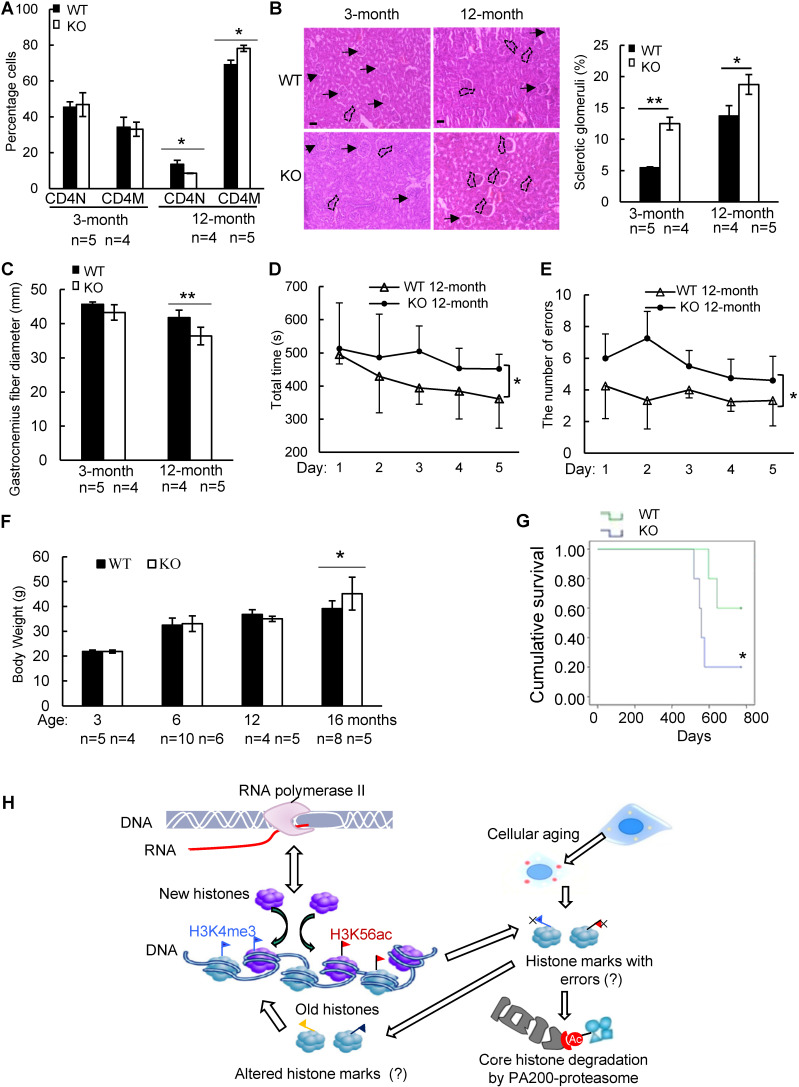
**Deletion of PA200 accelerates aging in mice. (A)** T cell subset analysis for naive CD4 (CD4N) and memory CD4 (CD4M) subsets in 3- and 12-month-old wild-type (WT) or PA200^-/-^ (KO) mice. * *p<*0.05; one-way ANOVA. **(B)** Hematoxylin-eosin stained kidney sections of 3- and 12-month-old mice. The phenotypes of renal sclerosis include interstitial inflammation (dashed area) and glomerular hypercellularity (as denoted by arrows) in KO mice (lower). The arrows denoted normal glomeruli in the wild-type mice (upper). Scale bar, 100 µm. The bar graph showed the percentage of sclerotic glomeruli from 12-month-old kidney sections. 40 glomeruli were scored for each animal. * *p<*0.05, ** *p<*0.01; one-way ANOVA. **(C)** The bar graph showed the gastrocnemius muscle fiber diameter of 3 and 12 months of age in WT and KO mice. ** *p<*0.01; one-way ANOVA. **(D)** The time in accomplishing eight successful performances of 8-arm maze test by 12-month-old WT or KO mice. * *p<*0.05; two-way ANOVA. **(E)** The number of errors made before eight successful performances in 8-arm maze test. Wild-type group: n=4, PA200^-/-^ group: n=5. * *p<*0.05; two-way ANOVA. **(F)** Body weights at different ages of wild-type and PA200 KO mice. * *p<*0.05; one-way ANOVA. **(G)** Lifespan in WT (n = 5) and PA200 KO (n = 5) mice. * *p<*0.05; one-way ANOVA. **(H)** A schematic showing a hypothetical model for the mechanism by which PA200 promotes the acetylation-dependent proteasomal degradation of the core histones during transcription and aging, and regulates deposition of the active transcriptional hallmarks, such as H3K4me3 and H3K56ac, and transcription, probably by degrading histones with abnormal marks. Data represent three independent biological replicates (mean ± SEM).

To further explore whether PA200 participates in regulation of aging, we analyzed the wild-type and PA200-deficient mice for distinct parameters of aging. The aging-related increase in memory T cells and reduction in naive T cells are important predictors of senescence 39. At 12-month old, female PA200^-/-^ mice had more memory and fewer naive CD4 T cells, however, these differences were not significant in the younger cohort (Figure 6A). Renal glomerulosclerosis usually occurs with aging 40. We analyzed interstitial inflammation and glomerular hypercellularity for glomerulosclerosis in the PA200-deficient mice. The results showed that the ratio of sclerotic glomeruli markedly increased at 3- or 12-month old in the PA200-deficient mice in comparison to the wild-type mice (Figure 6B). We also examined muscle loss, a hallmark of aging in both humans and rodents 41.

Gastrocnemius muscle fiber diameter declined with age more seriously in PA200^-/-^ mice than that in the wild-type mice. Unlike the wild-type mice, which showed an 8% decrease in gastrocnemius muscle fiber diameter between 3 and 12 months old, the PA200-deficient mice had a 15% decrease with age (Figure 6C), suggesting that PA200 deletion promotes muscle fiber atrophy. We then assessed anxiety-like behaviors in 12-month-old mice. PA200-deficient mice spent markedly more time and made more errors than the wild-type mice (Figure 6D-E), indicating that PA200 deficiency caused spatial learning defects in aging processes. Notably, the PA200-deficient mice at 16-month old were heavier than the wild-type counterparts, while younger mice showed no differences (Figure 6F). Finally, we examined mouse lifespan, and found that deletion of PA200 reduced mouse lifespan (Figure 6G). Taken together, these results suggest that deletion of PA200 leads to the aging-related deteriorations in mice.

## Discussion

The pattern of the histone post-translational modifications has been proposed as “histone code” 42. As the potential carriers of epigenetic code, histones have long been believed to be non-degradable during transcription. Indeed, parental histones can pass into the next generation of cells during DNA replication 43. A previous study has implicated the involvement of the ubiquitin-mediated protein degradation by the proteasome in the nucleosome turnover 10. Since the ubiquitin-proteasome pathway might regulate the nucleosome turnover indirectly, *e.g.*, by degrading histone chaperones that assist assembly of the nucleosome, the transcription-coupled degradation of histones has not yet been validated. We have previously shown that the PA200-containing proteasomes can promote the acetylation-dependent degradation of the core histones during somatic DNA repair and spermatogenesis 17. Moreover, Mandemaker et al. showed that the PA200-proteasome is required for the acetylation-dependent degradation of the core histones during DNA damage-induced replication stress 18. This study demonstrated that the PA200-containing proteasome promoted degradation of the core histones during transcription. Degradation of the histone variant H3.3, which is incorporated into chromatin during transcription, was much faster than that of its canonical form H3.1, which is incorporated during replication 27. As a support, we showed that the core histones were degraded in the G1-arrested cells. This degradation of the core histones could be suppressed by the transcription inhibitor, the proteasome inhibitor or deletion of PA200, but not by deletion of another proteasome activator PA28γ. Notably, the histone deacetylase inhibitor accelerated the degradation rates of H3 in general, especially its variant H3.3, while the mutations of the putative acetyl-lysine-binding region of PA200, which specifically binds the acetylated core histones *in vitro* 17, abolished histone degradation in the G1-arrested cells. This is consistent with our previous notion that acetylation is involved in degradation of the core histones 17. Thus, our results suggest that the core histones are degradable during transcription, and PA200 promotes their degradation in an acetylation-dependent manner.

The 19S complex of the 26S proteasome was shown earlier to function independently of the 20S catalytic particle, playing a direct and non-proteolytic role in RNA polymerase II-mediated transcription 44. But, it was suggested recently that the transcriptionally-relevant form of the proteasome is the canonical 26S complex 45, hinting that the degradation of certain proteins by the 26S proteasome might influence transcription indirectly. We have shown previously that the 19S particle is not directly involved in the degradation of the core histones, which were ectopically expressed 17. The situation could be different during transcription. Even during spermiogenesis, deletion of PA200 just retards the degradation of the core histones at step 11 of spermatogenesis, and the core histones are eventually degraded in the survived elongated spermatids and sperm in the PA200-deficient mice 17. As a result, the PA200-deficient mice are still fertile, though with the dramatically reduced fertility 46. This study demonstrates that PA200 regulates deposition of the transcriptionally-active histone marks, including H3K4me3 and H3K56ac. This deposition was positively correlated with the recruitment of RNA polymerase II onto chromatins, and inversely correlated with DNA methylation, which usually marks transcriptionally-inactive region in certain critical gene regions 47. Thus, these results further suggest that PA200 assures the deposition of proper histone marks.

Partial histone loss across the genome has been shown to be associated with aging in both yeast and human cells 20, 21. Transcription of various genes is altered during aging 48-50. The aging-related changes in transcription have been suggested to be caused by histone loss in yeast 9. This study showed that both PA200 and its yeast ortholog Blm10 promoted proteasomal degradation of the core histones during aging, and regulated transcription during cellular aging. Furthermore, deletion of either PA200 or Blm10 accelerated cellular aging. Notably, the PA200-deficient mice displayed a range of aging-related deteriorations, including immune malfunction, anxiety-like behaviors, and the reduced lifespan.

Although the cellular aging can prevent tumor development early in life, aging is also marked by an increase in tumorigenesis 48, 51, 52. Both aging and tumorigenesis are accompanied with the accumulation of abnormal histone marks 53, 54. PA200 deficiency caused abnormal transcription of many genes involved in cancers and altered deposition of H3K4me3 or H3K56ac on the genes related to cancer signaling, such as those in the p53, MAPK, and Ras signaling pathways. We speculate that these aging-related phenotypes of the PA200-deficient mice might be a consequence of the accumulated “old” histones with abnormal histone marks.

It has been a mystery how histone marks remain stable during transcription. Histones are frequently evicted from the nucleosome and then re-assembled in a transcription-coupled manner, a process referred to as nucleosome turnover 29. Histone exchange is generally highest at active promoters, where H3K4me3, H3K56ac, H3K9ac and H3K14ac usually accumulate 55. It can be imagined that certain mistakes in reassembling must happen during transcriptional nucleosome turnover, especially in the aged cells. Thus, the PA200/Blm10-mediated degradation of the core histones would probably eliminate the abnormally-assembled histone marks during transcription (Figure 6H). Our results also raise the possibility that the PA200-mediated degradation of the core histones facilitates nucleosome turnover. Although we could not exclude the possible involvement of other substrates of PA200 or an indirect role of PA200, the acetylation-dependent degradation of the core histones must play an important role in these PA200-related activities. Taken together, our results suggest that PA200 maintains the stability of histone marks during transcription and aging.

## Conclusions

The proteasome activator PA200 promotes the transcription-coupled degradation of the core histones, and plays an important role in maintaining the stability of histone marks during transcription and aging.

## Supplementary Material

Supplementary figures and tables.

## Abbreviations

Aha: azidohomoalanine
BRDL: bromodomain-like
ChIP: chromatin-immunoprecipitation
DEG: differentially expressed gene
DMEM: Dulbecco's modified Eagle's medium
DMR: differential methylation region
FBS: fetal bovine serum
GAHD: genome-wide analysis of histone degradation
MEF: mouse embryonic fibroblast
TSA: trichostatin A
TSS: transcriptional start site
TTS: transcriptional termination site
WGBS: whole-genome bisulfite sequencing

## References

[1] Campos EI, Reinberg D (2009). Histones: annotating chromatin. Annu Rev Genet.

[2] Ruthenburg AJ, Allis CD, Wysocka J (2007). Methylation of lysine 4 on histone H3: intricacy of writing and reading a single epigenetic mark. Mol Cell.

[3] Stejskal S, Stepka K, Tesarova L, Stejskal K, Matejkova M, Simara P (2015). Cell cycle-dependent changes in H3K56ac in human cells. Cell Cycle.

[4] Zhang B, Zheng H, Huang B, Li W, Xiang Y, Peng X (2016). Allelic reprogramming of the histone modification H3K4me3 in early mammalian development. Nature.

[5] Lessard JA, Crabtree GR (2010). Chromatin regulatory mechanisms in pluripotency. Annu Rev Cell Dev Biol.

[6] Portela A, Esteller M (2010). Epigenetic modifications and human disease. Nat Biotechnol.

[7] Qu Y, Yang Q, Liu J, Shi B, Ji M, Li G (2017). c-Myc is Required for BRAF(V600E)-Induced Epigenetic Silencing by H3K27me3 in Tumorigenesis. Theranostics.

[8] Hake SB, Allis CD (2006). Histone H3 variants and their potential role in indexing mammalian genomes: the “H3 barcode hypothesis”. Proc Natl Acad Sci U S A.

[9] Huang C, Zhu B (2014). H3.3 turnover: a mechanism to poise chromatin for transcription, or a response to open chromatin?. Bioessays.

[10] Maze I, Wenderski W, Noh KM, Bagot RC, Tzavaras N, Purushothaman I (2015). Critical Role of Histone Turnover in Neuronal Transcription and Plasticity. Neuron.

[11] Collins GA, Goldberg AL (2017). The Logic of the 26S Proteasome. Cell.

[12] Jiang TX, Zhao M, Qiu XB (2018). Substrate receptors of proteasomes. Biol Rev Camb Philos Soc.

[13] Navon A, Ciechanover A (2009). The 26 S proteasome: from basic mechanisms to drug targeting. J Biol Chem.

[14] Schmidt M, Haas W, Crosas B, Santamaria PG, Gygi SP, Walz T (2005). The HEAT repeat protein Blm10 regulates the yeast proteasome by capping the core particle. Nat Struct Mol Biol.

[15] Ustrell V, Pratt G, Gorbea C, Rechsteiner M (2005). Purification and assay of proteasome activator PA200. Methods Enzymol.

[16] Blickwedehl J, Agarwal M, Seong C, Pandita RK, Melendy T, Sung P (2008). Role for proteasome activator PA200 and postglutamyl proteasome activity in genomic stability. Proc Natl Acad Sci U S A.

[17] Qian MX, Pang Y, Liu CH, Haratake K, Du BY, Ji DY (2013). Acetylation-mediated proteasomal degradation of core histones during DNA repair and spermatogenesis. Cell.

[18] Mandemaker IK, Geijer ME, Kik I, Bezstarosti K, Rijkers E, Raams A (2018). DNA damage-induced replication stress results in PA200-proteasome-mediated degradation of acetylated histones. EMBO Rep.

[19] Hauer MH, Seeber A, Singh V, Thierry R, Sack R, Amitai A (2017). Histone degradation in response to DNA damage enhances chromatin dynamics and recombination rates. Nat Struct Mol Biol.

[20] Dang W, Steffen KK, Perry R, Dorsey JA, Johnson FB, Shilatifard A (2009). Histone H4 lysine 16 acetylation regulates cellular lifespan. Nature.

[21] Feser J, Truong D, Das C, Carson JJ, Kieft J, Harkness T (2010). Elevated histone expression promotes life span extension. Mol Cell.

[22] Deal RB, Henikoff JG, Henikoff S (2010). Genome-wide kinetics of nucleosome turnover determined by metabolic labeling of histones. Science.

[23] Kuma A, Hatano M, Matsui M, Yamamoto A, Nakaya H, Yoshimori T (2004). The role of autophagy during the early neonatal starvation period. Nature.

[24] Shechter D, Dormann HL, Allis CD, Hake SB (2007). Extraction, purification and analysis of histones. Nat Protoc.

[25] Smeal T, Claus J, Kennedy B, Cole F, Guarente L (1996). Loss of transcriptional silencing causes sterility in old mother cells of S. cerevisiae. Cell.

[26] Nguyen VT, Giannoni F, Dubois MF, Seo SJ, Vigneron M, Kedinger C (1996). *In vivo* degradation of RNA polymerase II largest subunit triggered by alpha-amanitin. Nucleic Acids Res.

[27] Tagami H, Ray-Gallet D, Almouzni G, Nakatani Y (2004). Histone H3.1 and H3.3 complexes mediate nucleosome assembly pathways dependent or independent of DNA synthesis. Cell.

[28] Dou Z, Xu C, Donahue G, Shimi T, Pan JA, Zhu J (2015). Autophagy mediates degradation of nuclear lamina. Nature.

[29] Dion MF, Kaplan T, Kim M, Buratowski S, Friedman N, Rando OJ (2007). Dynamics of replication-independent histone turnover in budding yeast. Science.

[30] Liu CL, Kaplan T, Kim M, Buratowski S, Schreiber SL, Friedman N (2005). Single-nucleosome mapping of histone modifications in S. cerevisiae. PLoS Biol.

[31] Meyers EA, Gobeske KT, Bond AM, Jarrett JC, Peng CY, Kessler JA (2016). Increased bone morphogenetic protein signaling contributes to age-related declines in neurogenesis and cognition. Neurobiol Aging.

[32] Brown JP, Wei W, Sedivy JM (1997). Bypass of senescence after disruption of p21CIP1/WAF1 gene in normal diploid human fibroblasts. Science.

[33] Agnihotri S, Wolf A, Picard D, Hawkins C, Guha A (2009). GATA4 is a regulator of astrocyte cell proliferation and apoptosis in the human and murine central nervous system. Oncogene.

[34] Collado M, Medema RH, Garcia-Cao I, Dubuisson ML, Barradas M, Glassford J (2000). Inhibition of the phosphoinositide 3-kinase pathway induces a senescence-like arrest mediated by p27Kip1. J Biol Chem.

[35] Perez VI, Bokov A, Van Remmen H, Mele J, Ran Q, Ikeno Y (2009). Is the oxidative stress theory of aging dead?. Biochim Biophys Acta.

[36] Krishnamurthy J, Torrice C, Ramsey MR, Kovalev GI, Al-Regaiey K, Su L (2004). Ink4a/Arf expression is a biomarker of aging. J Clin Invest.

[37] Wood JG, Rogina B, Lavu S, Howitz K, Helfand SL, Tatar M (2004). Sirtuin activators mimic caloric restriction and delay ageing in metazoans. Nature.

[38] Li B, Dewey CN (2011). RSEM: accurate transcript quantification from RNA-Seq data with or without a reference genome. BMC Bioinformatics.

[39] Miller RA (2001). Biomarkers of aging: prediction of longevity by using age-sensitive T-cell subset determinations in a middle-aged, genetically heterogeneous mouse population. J Gerontol A Biol Sci Med Sci.

[40] Schmitt R, Melk A (2017). Molecular mechanisms of renal aging. Kidney Int.

[41] Lexell J, Henriksson-Larsen K, Winblad B, Sjostrom M (1983). Distribution of different fiber types in human skeletal muscles: effects of aging studied in whole muscle cross sections. Muscle Nerve.

[42] Strahl BD, Allis CD (2000). The language of covalent histone modifications. Nature.

[43] Moazed D (2011). Mechanisms for the inheritance of chromatin states. Cell.

[44] Ferdous A, Kodadek T, Johnston SA (2002). A nonproteolytic function of the 19S regulatory subunit of the 26S proteasome is required for efficient activated transcription by human RNA polymerase II. Biochemistry.

[45] Geng F, Tansey WP (2012). Similar temporal and spatial recruitment of native 19S and 20S proteasome subunits to transcriptionally active chromatin. Proc Natl Acad Sci U S A.

[46] Khor B, Bredemeyer AL, Huang CY, Turnbull IR, Evans R, Maggi LB Jr (2006). Proteasome activator PA200 is required for normal spermatogenesis. Mol Cell Biol.

[47] Borgel J, Guibert S, Li Y, Chiba H, Schubeler D, Sasaki H (2010). Targets and dynamics of promoter DNA methylation during early mouse development. Nat Genet.

[48] Lopez-Otin C, Blasco MA, Partridge L, Serrano M, Kroemer G (2013). The hallmarks of aging. Cell.

[49] Harries LW, Hernandez D, Henley W, Wood AR, Holly AC, Bradley-Smith RM (2011). Human aging is characterized by focused changes in gene expression and deregulation of alternative splicing. Aging Cell.

[50] Nicholas A, de Magalhaes JP, Kraytsberg Y, Richfield EK, Levanon EY, Khrapko K (2010). Age-related gene-specific changes of A-to-I mRNA editing in the human brain. Mech Ageing Dev.

[51] Campisi J (2013). Aging, cellular senescence, and cancer. Annu Rev Physiol.

[52] Loaiza N, Demaria M (2016). Cellular senescence and tumor promotion: Is aging the key?. Biochim Biophys Acta.

[53] Lichtenstein AV, Kisseljova NP (2001). DNA methylation and carcinogenesis. Biochemistry (Mosc).

[54] Strub T, Ballotti R, Bertolotto C (2020). The “ART” of Epigenetics in Melanoma: From histone “Alterations, to Resistance and Therapies”. Theranostics.

[55] Kraushaar DC, Jin W, Maunakea A, Abraham B, Ha M, Zhao K (2013). Genome-wide incorporation dynamics reveal distinct categories of turnover for the histone variant H3.3. Genome Biol.

