# Supplementary material for: Proteasome activator PA200 maintains stability of histone marks during transcription and aging
Source: Theranostics. 2021 Jan 1;11(3):1458–72. doi: 10.7150/thno.48744 (PMC7738882; doi:10.7150/thno.48744)
Supplement: Supplementary file 1 — Supplementary figures and tables. [file thnov11p1458s1.pdf]

Supplementary Materials for

Proteasome activator PA200 maintains stability of histone marks during transcription and aging

Tian-Xia Jiang[1,*], Shuang Ma[1,*], Xia Han[2,*], Zi-Yu Luo[1], Qian-Qian Zhu[1], Tomoki Chiba[3], Wei Xie[4], Kui Lin[2] & Xiao-Bo Qiu[1]

* These authors contributed equally to this work

Corresponding authors: Xiao-Bo Qiu (xqiu@bnu.edu.cn) or Kui Lin (linkui@bnu.edu.cn).

This PDF file includes:

- Figures S1 to S9
- Tables S1 to S2
- Legends for Datasets S1 to S9
- Supplementary Methods
- Supplementary References

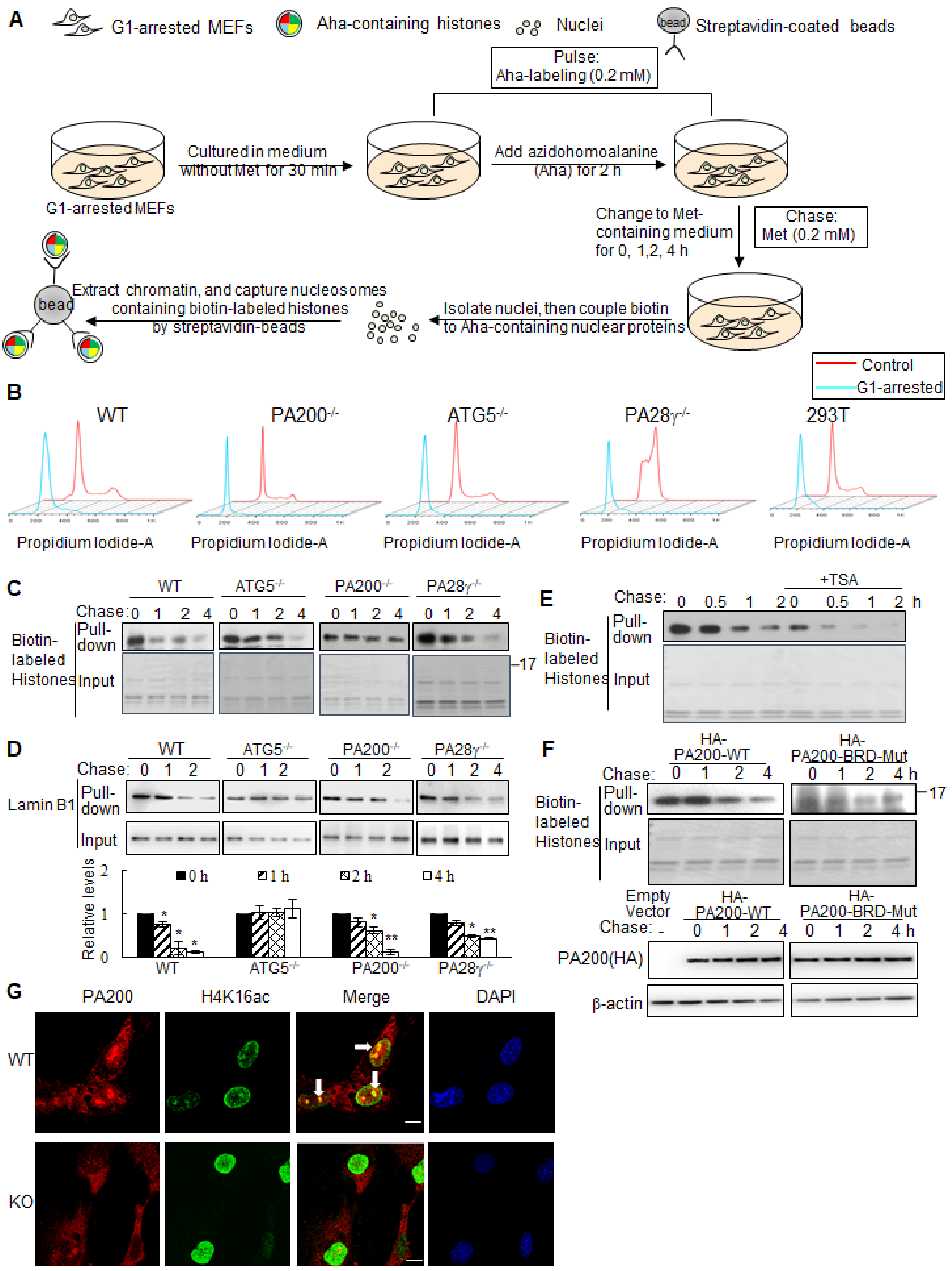

Figure S1. PA200 is essential for proteasomal degradation of core histones in G1-arrested cells. (A) Modified pulse-chase analysis of histone degradation during transcription. Aha was co-translationally

incorporated into proteins and subsequently ligated with biotin. Following chase in the regular medium with Met, old histones with Aha was affinity-purified with streptavidin for analysis of degradation. (B) MEF cells cultured for 24 h in the basal medium were treated with or without 0.5 mM minosine for 24 h. DNA content was analyzed by FACS analysis of cellular DNA with propidium iodide staining. 10000 cells were collected for analyzing in each group. (C, D) Aha-labeled histone (C) or lamin B1 (D) was captured by streptavidin-coupled beads and analyzed with streptavidin-HRP or anti-lamin B1 antibody by immunoblotting in the G1-arrested wild-type, PA200$^{-/-}$, ATG5$^{-/-}$, and PA28$^{-/-}$ MEF cells following the pulse-chase assay. The captured lamin B1 levels were quantified by densitometry (normalized to the corresponding input). * $p < 0.05$, ** $p < 0.01$, *** $p < 0.001$; one-way ANOVA. (E) The G1-arrested MEF cells were incubated in the absence or presence of 0.3 μM of TSA for 4 h, and histone degradation was analyzed as described in (C). (F) Histone degradation in the G1-arrested 293T transfected with either wild-type or mutant PA200 (PA200-BRD-Mut) with a HA-tag was analyzed as described in (C). The transfection efficiency was confirmed by detecting the levels of HA-tag. The biotin-labeled input histones in (E-F) were analyzed by Coomassie blue staining. Histones captured by streptavidin-coupled beads in (C, E, and F) were "pull-down" and analyzed by immunoblotting with their corresponding antibodies. The corresponding input histones, H3.3 and H3, in (C, E, and F) were used as loading controls. The input biotin-labeled histones were analyzed by Coomassie blue staining to show the equal loading. (G) Colocalization of PA200 with H4K16ac in G1-arrested MEF cells. PA200 was visualized with an antiserum against PA200 from rabbit (red), and H4K16ac was detected by a specific antibody from mouse (green), while nuclei were stained with DAPI. One of colocalization loci in each cell was indicated by an arrow. At least 20 cells were analyzed, and similar results were

obtained for almost all the cells. The PA200-deficient MEF cells (KO) served as controls for the specificity of the anti-PA200 antiserum. Data represent three independent biological replicates.

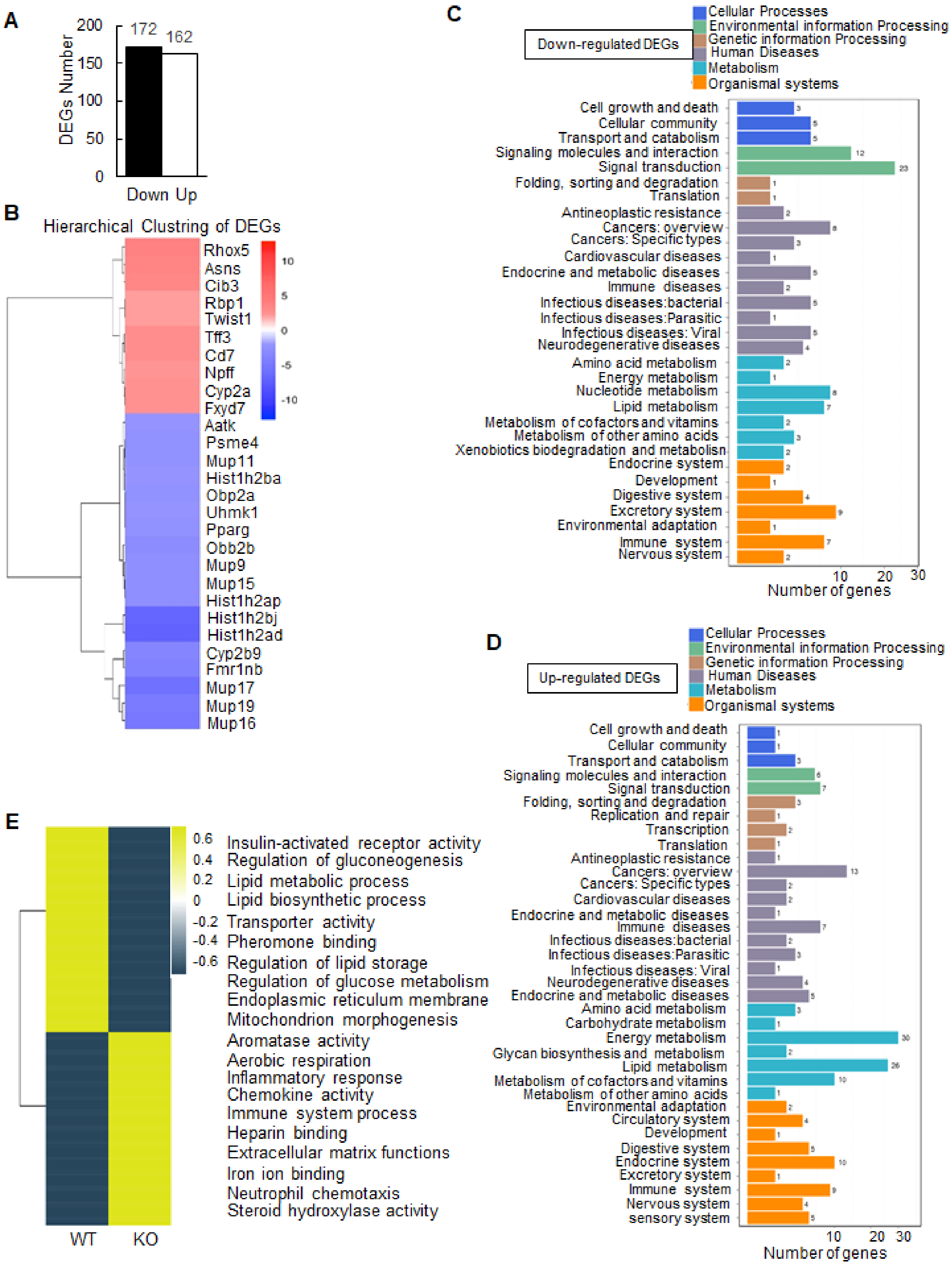

Figure S2. PA200 deletion influences gene expression as profiled by RNA-seq in mouse liver. (A) Numbers of the up-regulated and down-regulated genes in the PA200-deficient mouse liver

(normalized to the wild-type group). (B) Hierarchical clustering of 28 selected DEGs. All selected DEGs are up-regulated two to four folds or down-regulated two to seven folds in PA200-deficient mouse liver relative to wild-type liver. (C, D) KEGG pathway classification of the down-regulated (C) and up-regulated (D) DEGs following RNA-seq analyses of the PA200-deficient mouse liver in comparison to those of the wild-type liver. (E) Heat-map of all DEGs in the PA200-deficient mouse livers. DEGs were defined according to the combination of the absolute value of log2-ratio ≥1 and diverge probability≥0.8. Coloring indicates the log2-transformed fold change. DEGs were clustered into 20 major groups with enriched GO terms listed (right). All *p* values of GO Terms are <0.05.

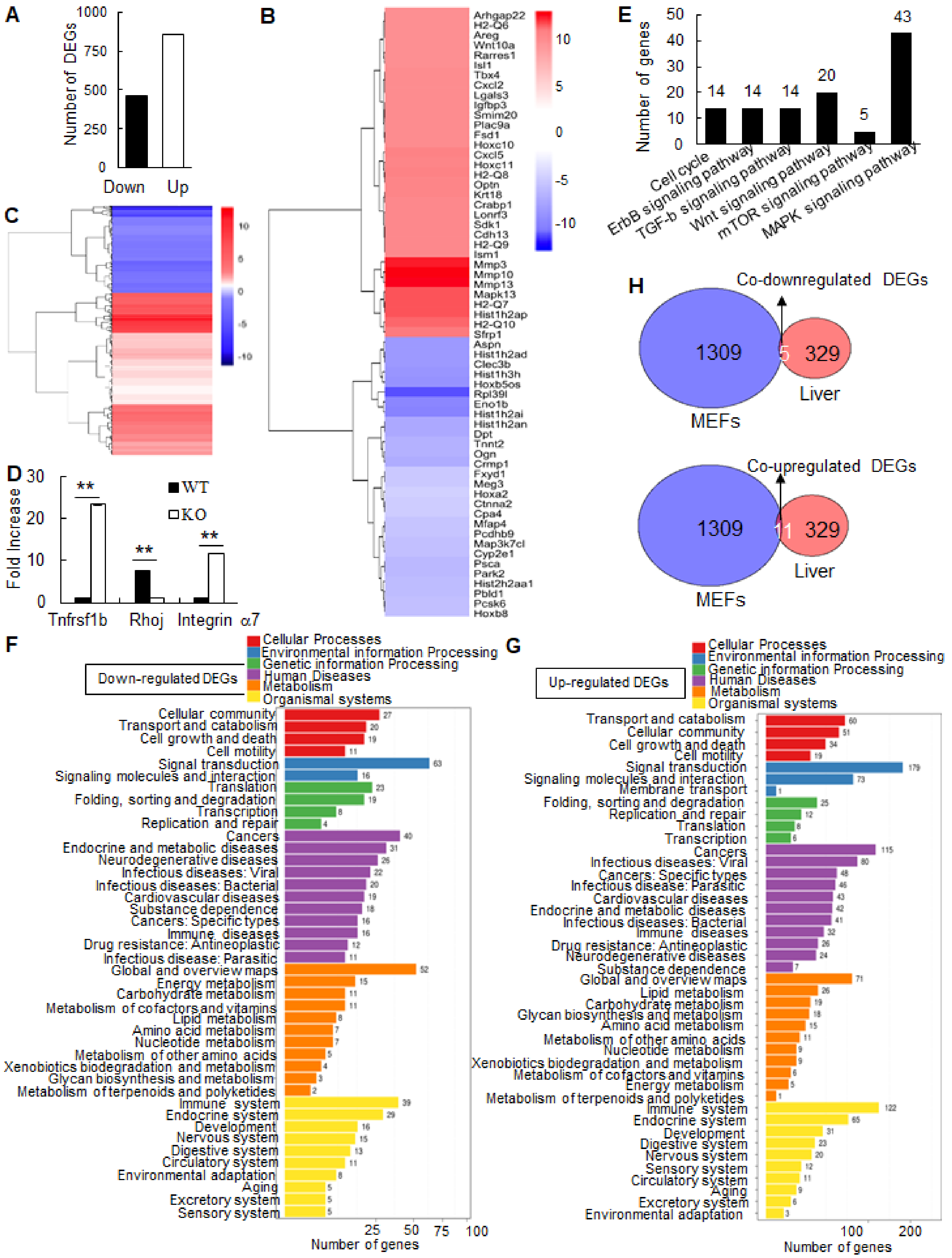

Figure S3. Gene expression profiling by RNA-seq in wild-type (WT) and PA200$^{-/-}$ (KO) MEFs. (A) Numbers of the up-regulated and down-regulated genes in the PA200-deficient MEF cells (normalized

to the wild-type group). (B) Hierarchical clustering of 61 selected DEGs. All selected DEGs are up-regulated ten to thirteen folds or down-regulated five to twelve folds in PA200$^{-/-}$ MEFs relative to WT MEFs. (C) Hierarchical clustering of intersection DEGs in the PA200-deficient MEF cells (normalized to the wild-type group). DEGs were defined according to the combination of the absolute value of log2-Ratio ≥1 and diverge probability≥0.8. Coloring indicates the log2 transformed fold change. (D) Quantitative PCR analysis of genes selected from the RNA-seq results, including up-regulated (Tnfrsf1 and Integrin α7) and down-regulated (Rhoj) genes. The bar graph represents three independent biological replicates, and the transcription levels were presented as relative folds of increase. ** $p <$ 0.01; one-way ANOVA. (E) KEGG classification into the aging-related pathways on DEGs in MEF cells. (F, G) KEGG pathway classification of down-regulated (F) and up-regulated (G) DEGs analyzed by RNA-seq in the PA200-deficient MEF cells (normalized to the wild-type group). (H) Co-up- or down-regulated genes in both the PA200-deficient livers and the G1-arrested PA200-deficient MEF cells.

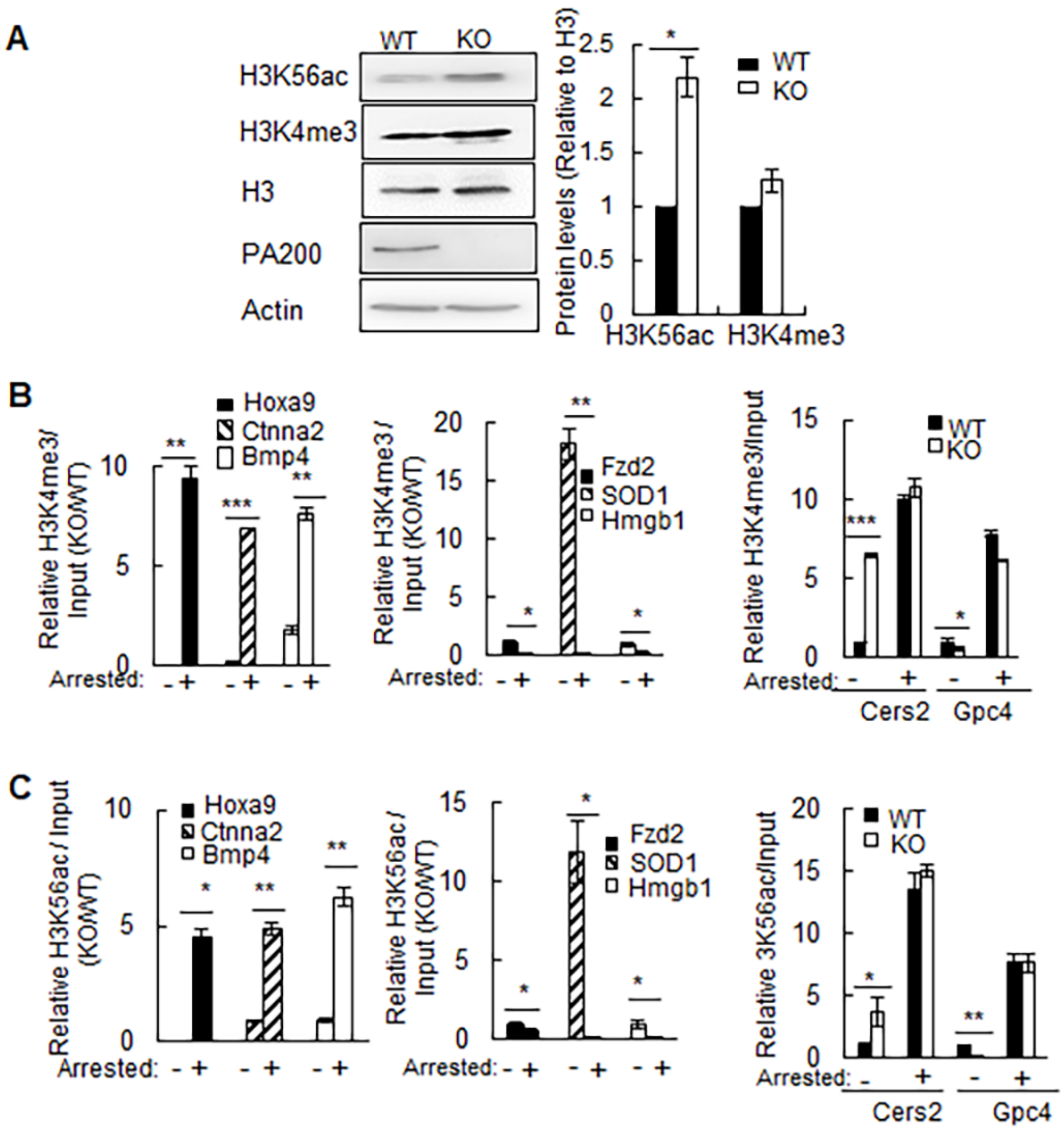

Figure S4. PA200 deletion affects protein levels of H3K56ac and H3K4me3, as well as the enrichments of the DEGs. (A) Immunoblotting analysis of H3K56ac or H3K4me3 levels in the PA200$^{+/+}$ (WT) and PA200$^{-/-}$ (KO) MEF cells. The levels of H3K56ac and H3K4me3 were quantified by densitometry (normalized to H3). * $p < 0.05$; one-way ANOVA. (B) ChIP-PCR analysis of the promoter levels of the up-regulated DEGs (*Hoxa9*, *Ctnna2*, *Bmp4*), down-regulated DEGs (*Fzd2*, *SOD1*, *HMGB1*), and not significantly changed genes (*Cers2* and *Gpc4*) on H3K4me3 in the G1-

arrested or non-arrested MEF cells. * $p < 0.05$, ** $p < 0.01$, *** $p < 0.001$; one-way ANOVA. (C) ChIP-PCR analysis of the promoter levels of the up-regulated DEGs (*Hoxa9, Ctnna2, Bmp4*), down-regulated DEGs (*Fzd2, SOD1, HMGB1*), and not significantly changed genes (*Cers2 and Gpc4*) on H3K56ac in the G1-arrested or non-arrested MEF cells. * $p < 0.05$, ** $p < 0.01$; one-way ANOVA. Data represent three independent biological replicates.

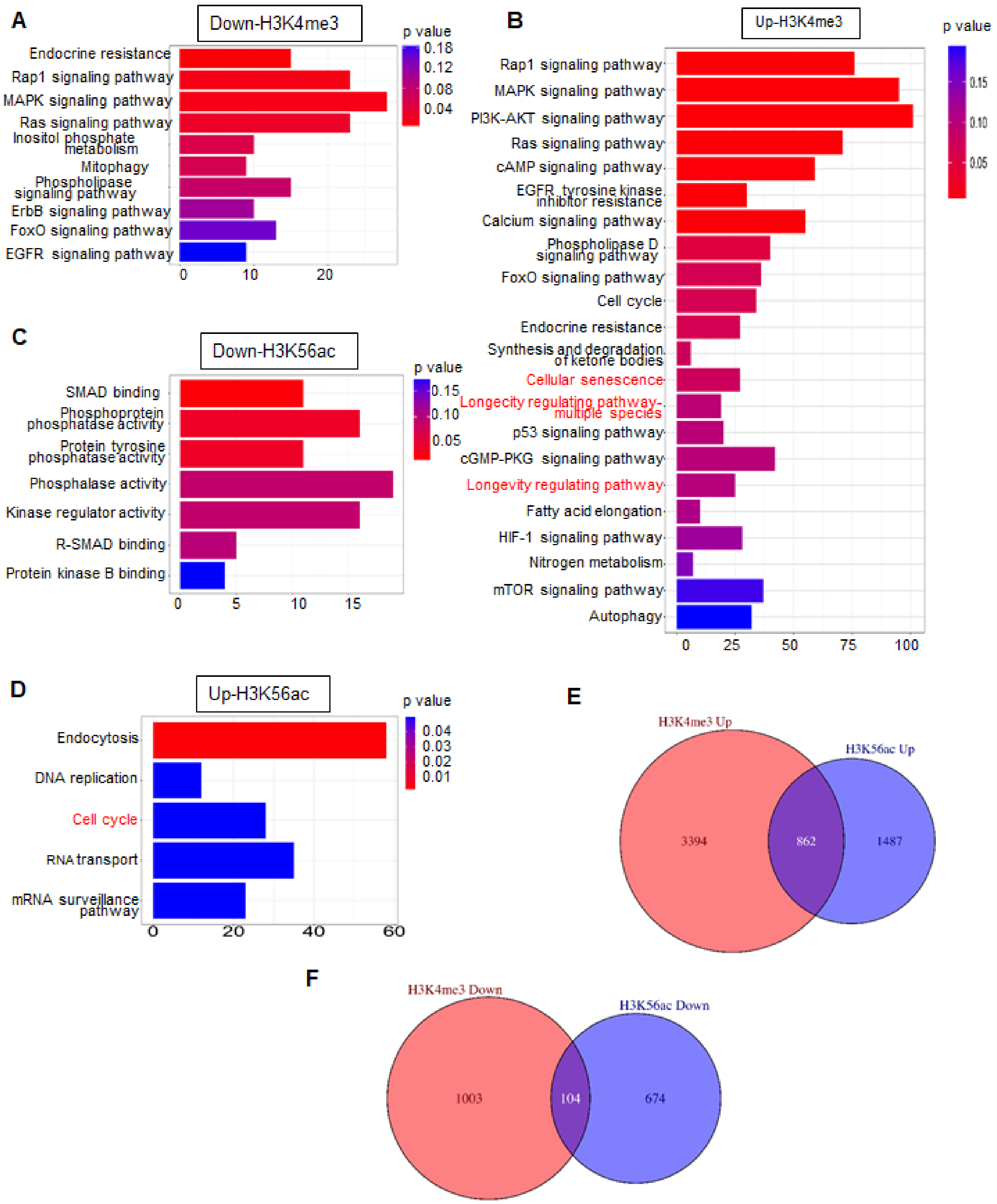

Figure S5. Analysis of H3K4me3 and H3K56ac-associated genes. (A, B) KEGG analysis of down-regulated (A) and up-regulated (B) regions-associated genes of H3K4me3 following ChIP-seq in the PA200-deficient MEF (normalized to the wild-type group). (C, D) KEGG analysis of genes within down-regulated (C) and up-regulated (D) H3K56ac-enriched regions in the PA200-deficient MEF

(normalized to the wild-type group). If their levels of H3K4me3 or H3K56ac in PA200$^{-/-}$ MEFs are higher than those in wild-type MEFs, these regions are defined as “Up-regulated” regions”. (E, F) Venn plots showing numbers of the common up-regulated (E) or down-regulated (F) genes between H3K4me3 and H3K56ac differential peaks in the PA200-deficient MEF cells (normalized to the wild-type group).

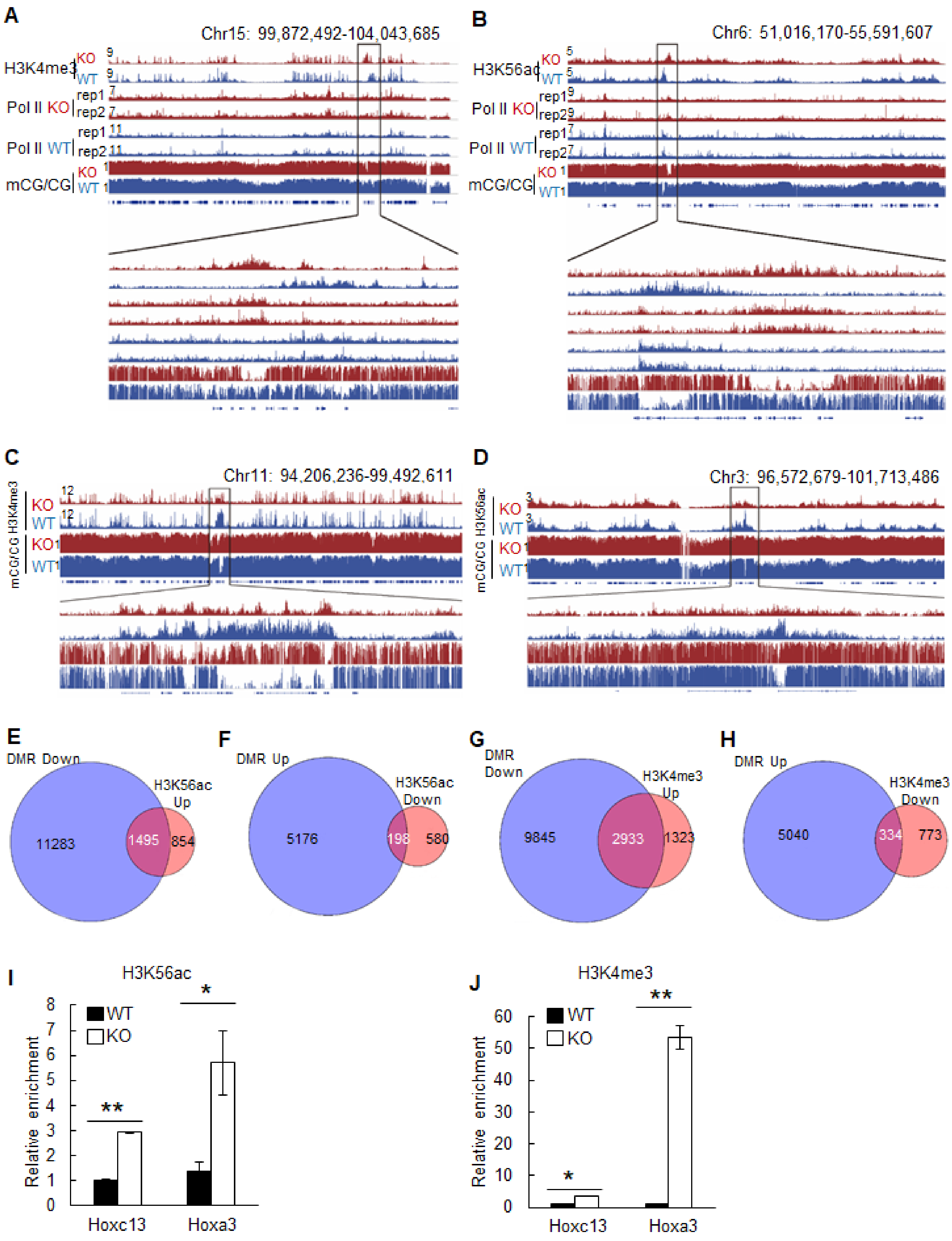

Figure S6. Changes of H3K56ac and H3K4me3 enrichments positively correlate with polymerase II recruitments, and inversely correlate with DNA methylation in certain gene regions. (A-D) The IGV

genome browser view of H3K4me3 (A, C) or H3K56ac (B, D) enrichment in WT and PA200$^{-/-}$ MEF chromosomes. The levels of DNA methylation and polymerase II are shown in parallel. (E, F) Venn plots showing numbers of genes that were up-regulated (E) or down-regulated (F) in H3K56ac differential peaks and inversely correlated with DNA methylation in certain gene regions in the PA200-deficient MEF cells. (G, H) Venn plots showing numbers of genes that were up-regulated (G) or down-regulated (H) in H3K4me3 differential peaks and inversely correlated with DNA methylation in certain gene regions in the PA200-deficient MEF cells. (I-J) Quantitative PCR analysis of the genes with the coordinated association with both RNA polymerase II and H3K56ac or H3K4me3 (including Hoxc13 and Hoxa3). Three independent experiments were carried out, and results represent the relative enrichment of average value for each genomic segment. * $p < 0.05$, ** $p < 0.01$; one-way ANOVA.

A

Sample	Bisulfite conversion rate (%)	Coverage of CpG sites (Mb)	Average CpG methylation (%)	Average read depth of CpG sites
WT	99.833	364.3	66.04	7.7
KO	99.877	344.3	68.06	8.0

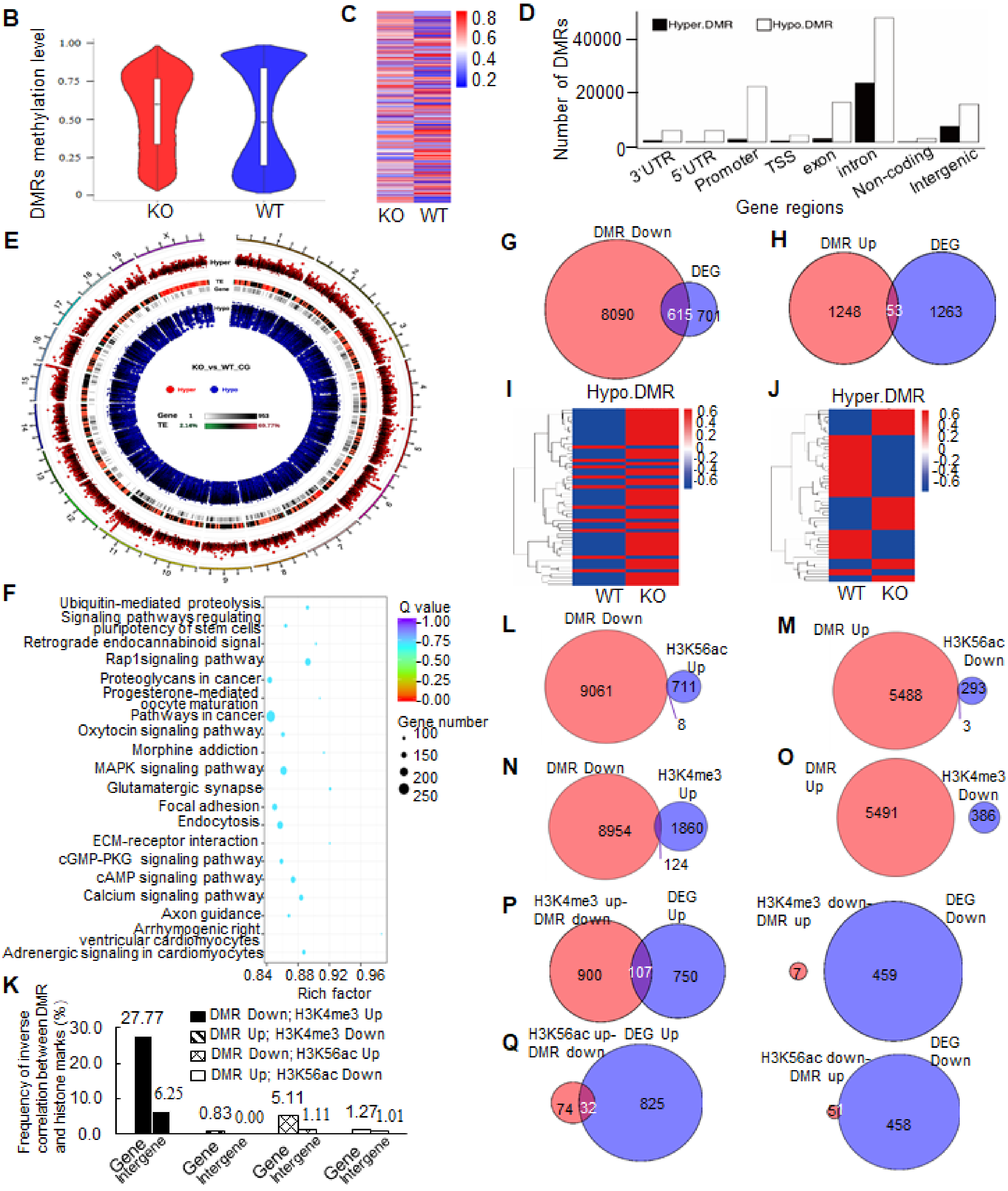

Figure S7. Differential methylome analysis in the wild-type and PA200$^{-/-}$ MEF cells. (A) Summary of WGBS analysis from the wild-type (WT) and PA200$^{-/-}$ (KO) MEF cells. (B) Violin plots showing the

distributions of DNA methylation levels in WT and PA200$^{-/-}$ MEFs. (C) Heatmap of methylation levels within CG DMRs in WT and PA200$^{-/-}$ MEF cells. (D) Distributions of CG DMRs gene regions in the PA200-deficient MEF cells (normalized to the wild-type group). (E, F) Venn plots showing gene numbers of hypo-DMRs (E) or hyper-DMRs (F) and DEGs in PA200-deficient MEF cells. **(**G, H**)** Hierarchical clustering of the hypo-DMRs-related DEGs (G) or hyper-DMRs-related DEGs (H) in the PA200$^{+/+}$ and PA200$^{-/-}$ MEF cells. (I) The frequency of inverse correlation between DMR and peaks of histone marks. Frequency= the peak number of inverse correlation / total peak number of histone marks in gene regions or intergene regions. Gene and intergene regions were defined by the HOMER (v4.9.1). The gene region includes TSS, promoter, intron, and exon. (J) (K) Venn plots showing peak numbers of H3K56ac and DNA methylation that were upregulated (J) or downregulated (K) in the intergene regions of the PA200-deficient MEF cells. **(**L, M**)** Venn plots showing peak numbers of H3K4me3 and DNA methylation that were up-regulated (L) or down-regulated (M) in the intergene regions of the PA200-deficient MEF cells. (N) Venn plots showing numbers of the DEGs and genes that corresponds to the "inverse correlation of H3K4me3 and DMRs" regions in the PA200-deficient MEF cells. (O) Venn plots showing numbers of the DEGs and genes that corresponds to the "inverse correlation of H3K56ac and DMRs" regions in the PA200-deficient MEF cells. (P) Circular representation of the genome-wide distribution of CG DMRs in the PA200-deficient MEF cells (normalized to the wild-type group). This visualization was generated using the Circos software58. Hyper, hyper-methylated DMRs; Hypo, hypo-methylated DMRs. (Q) KEGG pathway enrichment of CG DMRs genes in the PA200-deficient MEF cells (normalized to the wild-type group).

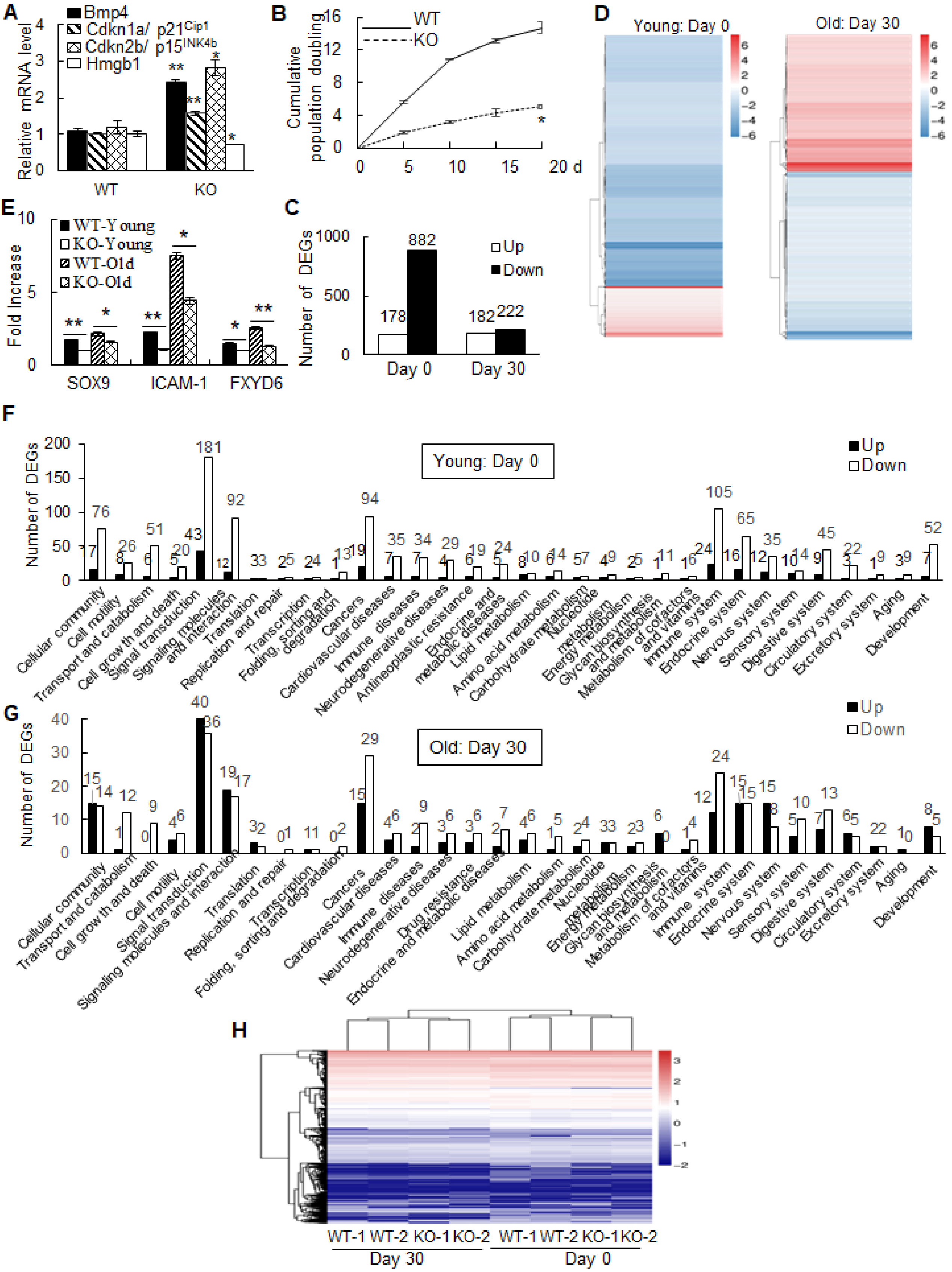
A
Bmp4
Cdkn1a/ p21Cip1
Cdkn2b/ p15INK4b
Hmgb1
Relative mRNA level
WT
KO
B
WT
KO
Cumulative population doubling
20 d
D
Young: Day 0
Old: Day 30
E
WT-Young
KO-Young
WT-Old
KO-Old
Fold Increase
SOX9
ICAM-1
FXYD6
C
Number of DEGs
Up
Down
882
178
182
222
Day 0
Day 30
F
Young: Day 0
Number of DEGs
Up
Down
Cellular community
Cell motility
Transport and catabolism
Cell growth and death
Signal transduction
Signaling molecules and interaction
Translation
Replication and repair
Transcription
Folding, sorting and degradation
Cancers
Cardiovascular diseases
Immune diseases
Neurodegenerative diseases
Antineoplastic resistance
Endocrine and metabolic diseases
Lipid metabolism
Amino acid metabolism
Carbohydrate metabolism
Nucleotide metabolism
Energy metabolism
Glycan biosynthesis and metabolism
Metabolism of cofactors and vitamins
Immune system
Endocrine system
Nervous system
Sensory system
Digestive system
Circulatory system
Excretory system
Aging
Development
G
Old: Day 30
Number of DEGs
Up
Down
Cellular community
Transport and catabolism
Cell growth and death
Cell motility
Signal transduction
Signaling molecules and interaction
Translation
Replication and repair
Transcription
Folding, sorting and degradation
Cancers
Cardiovascular diseases
Immune diseases
Neurodegenerative diseases
Drug resistance
Endocrine and metabolic diseases
Lipid metabolism
Amino acid metabolism
Carbohydrate metabolism
Nucleotide metabolism
Energy metabolism
Glycan biosynthesis and metabolism
Metabolism of cofactors and vitamins
Immune system
Endocrine system
Nervous system
Sensory system
Digestive system
Circulatory system
Excretory system
Aging
Development
H
WT-1 WT-2 KO-1 KO-2 WT-1 WT-2 KO-1 KO-2
Day 30
Day 0

Figure S8. Deletion of PA200 changes gene expression during aging. (A) Quantitative PCR analysis of 4 selected aging markers in mouse livers. (B) Growth curve for the cumulative population doubling of primary wild-type (WT) and PA200-deficient (KO) MEF cells. (C) Numbers of the up-regulated and down-regulated genes in the PA200-deficient primary MEF cells (normalized to the wild-type group). (D) Hierarchical clustering of intersection DEGs in the PA200-deficient primary MEF cells (normalized to the wild-type group). (E) Quantitative PCR analysis of genes (SOX9, ICAM-1 and FXYD6) selected from RNA-seq results of primary MEF cells cultured for 0 (young) or 30 (old) days. The transcription levels were presented as relative folds of increase. Data represent three independent biological replicates (** $p < 0.01$, * $p < 0.05$; one-way ANOVA). (F, G) KEGG pathway classification of up-regulated and down-regulated DEGs analyzed by RNA-seq in young (F) or old (G) PA200-deficient primary MEF cells (normalized to the wild-type group). (H) Expression heatmap of transcription factor TF genes in the wild-type and PA200-deficient primary MEFs. Coloring indicates the log2 transformed fold change.

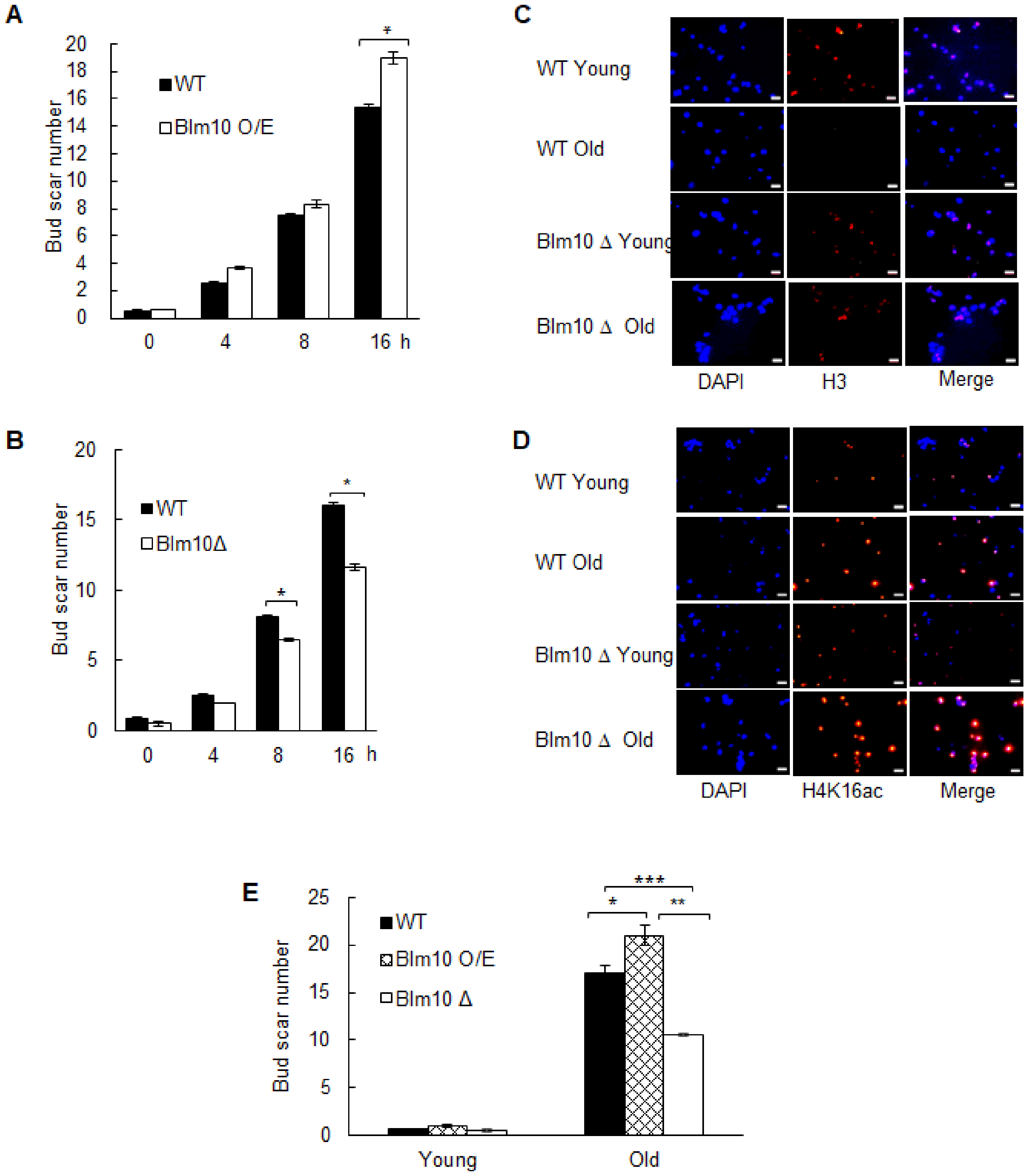

Figure S9. Effects of PA200/Blm10 on the levels of H3, H4 and H4K16ac during cellular aging. (A, B) Scar number in the young or old wild-type, Blm10-overexpressing (Fig.5E) and Blm10-deficient (Fig. 5F) yeast. More than 20 cells were measured in each group. * $p < 0.05$, ** $p < 0.01$; one-way ANOVA. (C, D) Immunofluorescent images of histone H3 (C) and H4K16Ac (D) in the young or old

wild-type and Blm10-deficient yeast. More than 20 cells were measured in each group. Scale bar, 5 μm. (E) Bud scar numbers in yeast as in Fig. 5D. Young: 0 hour; Old: 80 hours. Data represent three independent biological replicates (* $p < 0.05$, ** $p < 0.01$, *** $p < 0.001$; one-way ANOVA.).

Table S1. Aging-related DEGs in PA200$^{-/-}$ (KO) and WT MEF cells / Livers

	Upregulated aging genes			Downregulated aging genes		
	Gene Name	ID Number	log2Ratio (KO/WT)	Gene Name	ID Number	log2Ratio (KO/WT)
MEF cells	Acsl1	14081	0.97105	Bmi1	12151	-0.9523
	Adam10	11487	1.1754	Brca2	12190	-1.11145
	Ago2	239528	0.972634	Cacna1g	12291	-1.03264
	Ankrd11	77087	0.963493	Cacna1a	12286	-2.58114
	Apoe	11816	1.312121	Ccdc92	215707	-2.32193
	Bhlhe40	20893	0.995111	Ccnd1	12443	-1.24946
	Bmp4	12159	3.395806	CD28	12487	-2.70044
	Brd4	57261	0.972746	Cfl1	12631	-0.96447
	Bhlhe40	20893	0.995111	Nos3	18127	-0.95899
	Cdkn1a/ P21^{Cip1}	12575	1.08458	Cox5b	12859	-0.9641
	Cdkn2a/ p16^{INK4a}	12578	1.02683	Cxcl12	20315	-1.86064
	Cdkn2b/ p15^{INK4b}	12579	0.994573	Epb41l3	13823	-0.98135
	Cdv3	321022	1.02952	Fkbp11	66120	-1.15039
	Cebpb	12608	1.127962	Fxn	14297	-0.98007
	Clk1	12747	0.9486608	Hist1h2bk	319184	-6.06609
	Crebbp	12914	0.986413	HMGB1	15289	-0.951504
	Dusp12	80915	0.977664	Il2ra	16184	-1.29956
	Elavl1	15568	0.977701	Lsm4	50783	-1.0951
	Ereg	13874	3.796848	Mapt	17762	-1.17306
	Hmox1	15368	1.042784	Nap1l2	17954	-1.16993
	Il6	16193	2.472956	Nos3	18127	-0.95899
	Icos	50723	8.129283	Park2	50873	-6.56533
	Igf1	16000	1.850857	Phb	18673	-0.96328
	Kazn	71529	5.686501	Prdx2	21672	-1.51461
	Kcnj2	16518	3.514573	Psme4	103554	-1.29889
	Mcm7	17220	0.987018	Slit1	20562	-1.22239
	Mmp2	17390	8.400518	Sirt1	64383	-1.32991
	Morc2a	74522	0.983955	Rgn	19733	-8.00282
	Mtor	56717	1.161428	Satb1	20230	-4.33985
	Nfkb2	18034	1.182677	Sod1	20655	-1.03998
	Npc1	18145	1.286464	Tnfrsf21	94185	-2.66297
	Plekhm1	353047	0.952412	Ucp2	22228	-4.00505
	Sdccag3	68112	0.980982	Wars	70560	-0.96223
	Tgfb1	21803	2.940894			
Livers	Atf3	11910	1.326981	Aatk	11302	-2.01238
	Bmp4	12159	1.136285	Atxn1	20238	-1.045
	Casp1	12362	1.050305	Cyp2b10	13088	-1.25764
	Daam2	76441	1.030762	Cd36	12491	-1.72374
	Esm1	71690	1.765535	Clstn3	232370	-1.98185
	Fstl1	14314	1.166972	Frat1	14296	-1.04862
	Gstp2	14869	1.292151	Lmnb1	16906	-1.0346
	Lor	16939	1.067279	Mup1	17840	-1.27419
	Lpl	16956	1.468883	Phospho1	237928	-1.00221
	Mt1	17748	1.265795	Psme4	103554	-2.28972
	Mt2	17750	1.393635	Pparg	19016	-2.2158
	Npff	54615	2.398426	Rcor1	217864	-1.05561
	Oas1a	246730	1.079289	S100a6	20200	-1.13227
	Rbp1	19659	2.01828	Tnnt2	21956	-1.55325
	Sox17	20671	1.0199			
	Twist1	22160	1.97904			
	Xaf1	327959	1.043903			

Supplementary Methods

Mice. The PA28γ-deficient mice (C57BL/6N) were kindly provided by Drs. Lance Barton and Xiaotao Li. The PA200-deficient mice (C57BL/6N) were generated with the CRISPR/Cas9 technique by Beijing Biocytogen Co., Ltd (Beijing, China). In brief, PA200$^{-/-}$ mice were generated by nonhomology end joining, which was induced by 2 double-strand break repairs after introduction of 2 single-guide RNAs with Cas9. Two single-guide RNAs were designed to target a region upstream of intron 42 and downstream of 3'UTR, respectively. Different concentrations of Cas9 mRNA and single-guide RNAs were mixed and co-injected into the cytoplasm of 1-cell-stage fertilized eggs to generate chimeras. Polymerase chain reaction genotyping and sequencing revealed that some pups carried deletions of about 21-kb spanning 2 single-guide RNA target sites, removing the PA200 amino acid 1606-1843.

For genotyping, DNA was extracted from tip of the tail and analyzed by PCR with the primers: forward primer 5'-TGTTCACCACTGAAGTATAGGAACTCA, reverse primer 5'-GCTGGAGT ATTCTTCCCTT GGGAGT (for PA200$^{-/-}$ mice); forward primer 5'-AGCTGTGAAGATCATG AGCTATAGTAGT, reverse primer 5'-TCTGTGAGTCTGAAGCCTGGTCTAA (for wild-type mice).

The animals' care was in accordance with institutional guidelines. Animal care, surgery and handling procedures were performed according to regulations established by the Ministry of Science and Technology of the People's Republic of China ([2006] 398) and approved by the Ethic and Animal Welfare Committee of College of Life Science, Beijing Normal University.

Antibody information. Antibodies against the histone H3 (1:1000, Abcam, #ab1791), H4 (1:3000, EMD Millipore, #05-858), H3.1 (1:1000, Active Motif, #61629), H3.3 (1:1000, Abcam, #ab176840), PA200 (1:500, Abcam, #ab181203), HMGB1 (1:1000, Cell signaling technology, #6893), Lamin B1 (1:5000, Abcam, #ab133741), Rpb1 (1:2000, Abcam,#ab76123), HA (1:2000; Santa Cruze, sc7392), PA28γ (1:3000, Enzo,

PW8190), streptavidin-HRP (1:5000, ZSGB-BIO, #ZB-2404), and β-actin (1:5000, Sigma-Aldrich, #A5441) were used as primary antibodies to detect the corresponding proteins. Peroxidase-conjugated anti-mouse IgG (1:5000,ZSGB-BIO,#ZB-5305), anti-rat IgG (1:4000, ZSGB-BIO, #ZB-2307) or anti-rabbit IgG (1:3000, ZSGB-BIO, #ZB-5301) was used as secondary antibody. Antibodies against H3K56ac (Abcam, #ab76307), H3K4me3 (Abcam, #ab8580) and RNA polymerase II (Abcam, #Ab5095) were used for ChIP-seq assay.

RNA-seq preparation and data processing. RNA samples were collected from G1-arrested MEF cell lines or mouse livers using TRIZOL reagent with two biological replicates, and sequenced on Illumina BGISEQ-500 at Beijing Genomic Institution (BGI, Shenzhen, China; http://www.genomics.org.cn). Clean-tags were aligned to the mm10 reference genome. For gene expression analysis, the matched reads were calculated and then normalized to RPKM using RESM software [1]. Differential expression of genes (DEGs) were defined by the NoISeq method according to the following criteria: diverge probability ≥0.8 and the absolute value of log2-Ratio ≥1(fold of change ≥2) [2]. Since DE probability equals to 1-FDR, 1-probability can be used as an adjusted P-value [2]. Gene Ontology (GO) and pathway annotation and enrichment analyses were based on the Gene Ontology Database (http://www.geneontology.org/) and KEGG pathway database (http://www.genome.jp/kegg/), respectively. The software Cluster and Java Tree view were used for hierarchical cluster analysis of gene expression patterns [3, 4]. DEGs were defined according to the combination of the absolute value of log2-Ratio ≥1 and diverge probability≥0.8. Coloring indicates the log2 transformed fold change. We performed hierarchical cluster analysis of DEGs using the heatmap method in R version 3.3.3. We measured the distance between genes using the agglomeration method (Ward.D2).

Quantitative PCR. The Transcriptor First Strand cDNA synthesis Kit (Roche) was used for cDNA synthesis from total RNA according to manufacturer's instructions. Quantitative PCR was performed with an ABI 7500 Real-Time PCR System (Applied Biosystems) in technical duplicates from two biological replicates. Gapdh was set as control. Relative expression values were calculated using the ΔCt method. The primer sequences were listed in Table S2.

Table S2. Primers used in this study.

Gene	Species	Primer sequences （5' to 3'）
**p21^{Cip1}**	Mouse	F: CAGACCAGCCTGACAGATTTC; R: GGCACTTCAGGGTTTTCTCTT
**p15^{INK4b}**	Mouse	F: CGACCCTGCCACCCTTACCA; R: TGCTCTTCAGCCAAGTCTACCG
**Hmgb1**	Mouse	F: ACCCGGATGCTTCTGTCAAC; R: GGTGCATTGGGGTCCTTGAA
**Bmp4**	Mouse	F: CGAGCCAACACTGTGAGGAG; R: CCGAGGAGATCACCTCATTC
**SOD1**	Mouse	F: CCAGTGCAGGACCTCATTTT; R: TCCCAGCATTTCCAGTCTTT
**AMPKα1**	Mouse	F: CTCACCTCCTCCAAGTTATT; R: TCAGATGGGCTTATACAGC
**EGFR**	Mouse	F: GACCTTCACATCCTGCCAGT; R: GCATGGAGGTCAGTCCAGTT
**Adam10**	Mouse	F:GTGCCAGTACAGGCTCTTTGC;R:CACAGTAGCCTCTGAAGTCATTACATG
**Ccnd2**	Mouse	F: GTGCCAGTACAGGCTCTTTGC; R: GCCAGGTTCCACTTCAGCTTA
**Fzd2**	Mouse	F:ATTTAGTGGACATGCAGCGATTTC;R:AGCAGGAAGGATGTACCGATGAA
**ApoE**	Mouse	F:TCCTGTCCTGCAACAACATCC; R:AGGTGCTTGAGACAGGGCC
**Tnfrsf1b**	Mouse	F: CGGGCCAACATGCAAAAGTC; R: CAGATGCGGTTCTGTTCCC
**Rhoj**	Mouse	F:CGGCTGCAATGGACATGAG; R:GGCACGTATTCCTCTGGGAAG
**Integrin α7**	Mouse	F: AAG AGG GGT GCT GAG GTG AAA; R: CAA GGT CAA GTC TCC GGC TG
**SOX9**	Mouse	F: ACGTGGACATCGGTGAACTGA;R: GGCAAGTATTGGTCAAACTCATTGA

**ICAM-1**	Mouse	F: CGCTGTGCTTTGAGAACTGT; R: AGGTCCTTGCCTACTTGCTG
**FXYD6**	Mouse	F: ATGGAGACGGTGCTGGTCCT; R: TCAGTTCTCTGCCTTCTGGG
**Gapdh**	Mouse	F: AACTTTGGCATTGTGGAAGG; R: GGATGCAGGGATGATGTTCT
**SOD1 (ChIP-PCR)**	Mouse	F: TCCATCCTTTTGTCTCAG; R: GATACTTCATTCCTCCTTAA
**Fzd2 (ChIP-PCR)**	Mouse	F: CTGGGTAGAGGAGGGTGGGG; R: TCGGACTGAGGGTCGGAGAC
**Hmgb1 (ChIP-PCR）**	Mouse	F: ATTGGGGTCCTTGAACTT; R: GATATGGCAAAGGCTGAC
**Bmp4 (ChIP-PCR）**	Mouse	F: CAGCCCAATTTCCACAAC; R: TTCAACGCAGCGACTACA
**Hoxa9 (ChIP-PCR）**	Mouse	F: AATCTGTTGGTCGCTCCT; R: CAAATCGCATTCTCACTCTAC
**Ctnna2 (ChIP-PCR）**	Mouse	F: CTTTGCTGGCAGCCCTGAG; R: GTGGGTCGGTGGGAGTTTCT
**Gpc4 (ChIP-PCR）**	Mouse	F: CGCTCCGCTCGTGAGTGTT; R: GGTGCGGACCCTGACGGAC
**Cers2 (ChIP-PCR）**	Mouse	F: CCTCCTTCAGCAACTCCA; R: TGACAGGAGAAAGGTGGC

Analysis of ChIP-seq enrichment patterns. The ChIP-seq average signals at promoters (±2.5 kb) of DEGs were computed by ngs.plot, and gene expression levels (RPKM) were used in the analysis. The correlation between ChIP-seq enrichment at promoters and gene expression was shown in heat maps.

For different ChIP-Seq data (such as H3K4me3 in the wild-type and PA200-deficient MEF cells), we used MAnorm to statistically compare the quantitative binding differences and used M values to classify the upregulated or downregulated regions. For the identified up/down-regulated regions of H3K4me3/H3K56ac, we annotated their related genes as enrichment by Homer. The ChIP-seq (polymerase II or histone marks) enrichment patterns at the corresponding genes were calculated by ngs.plot.

Whole-genome bisulfite sequencing (WGBS) for DNA methylation. The genome DNA was isolated from MEF cells using TIANamp Genomic DNA Kit (TIANGEN, #DP304) according to manufacturer's protocol. The purified DNA was then directly proceeded to quality inspection, library construction and sequencing at the Novogene Bioinformatics Institute (Beijing, China) on an Illumina HiSeq 2000/2500 platform. Briefly, approximately 5.2 μg of genomic DNA spiked with 26 ng of lambda DNA was fragmented by sonication to 200-300 bp with Covaris S220, followed by end repair and adenylation. The sonicated DNA was then ligated with cytosine-methylated barcodes. These DNA fragments were treated with bisulfite using EZ DNA Methylation-GoldTM Kit twice. The resulting single-strand DNA fragments were amplified by PCR using KAPA HiFi HotStart Uracil + ReadyMix (2×). Qubit® 2.0 Fluorometern was selected to quantify the library concentration. The insert size was assessed on an Agilent Bioanalyzer 2100 system. Subsequently, 125 bp single-end reads were generated. Image analysis and base calling were performed with Illumina CASAVA pipeline. Finally, 125 bp paired-end reads were generated. Clean reads were obtained as previously described [5]. The reference genome was transformed into the bisulfite-converted version (C-to-T and G-to-A converted) and indexed by Bowtie 2. Clean reads were also transformed into the fully bisulfite-converted version and then aligned to the above converted version of the genome. Differentially methylated regions (DMR) were analyzed by DSS software, which is based on beta-binomial distribution, and the related genes of DMRs were annotated. For DMR, we identified the upregulated/hyper-DMR if the mean methylated value of KO sample is larger than that of WT sample, and *vice versa* for the downregulated/hypo-DMR. For analysis of genome-wide methylation, each chromosome was classified into several bins with equal sizes, and the methylation level of each bin was calculated as the proportion (mC counts / mC counts + umC counts). The high methylation level of genome was calculated as the ratio of the number of high-methylated (>0.5) bins to the number of all bins.

KEGG enrichment analysis of DMR-related genes was implemented by the GO-seq R package [6] in which gene length bias was corrected. GO terms with corrected P-value less than 0.05 were considered significantly enriched by DMR-related genes. We used KOBAS software [7] to test the statistical enrichment of DMR-related genes in KEGG pathways.

ChIP-seq preparation and data processing. About 5×10^7 cells were used for each ChIP-Seq assay. Two micrograms of either histone H3K4me3 antibody or histone H3K56ac antibody were used for each immunoprecipitation reaction. The sequence libraries were generated using the purification kit (CST, #14209) for in-depth whole-genome DNA sequencing by the Illumina HiSeq 2500 platform (BGI, Shenzhen, China; http://www.genomics.org.cn,). ChIP–seq reads were aligned to the mouse reference genome (mm10) using HISAT2 (version 2.0.3). All unmapped reads, non-uniquely mapped reads and PCR duplicates were removed by SAM tools (version 1.3.1). To call peaks, we used MACS2 with the parameters –bdg –broad –nomodel and –SPMR to normalize each sample by sequencing depth, and used subcommand bdgcmp with parameter –m FE to get noise-subtracted tracks. After identifying peaks by MACS2 in each ChIP-seq sample, we quantitatively compared peaks of ChIP-Seq samples using MAnorm, which calculates M value and P-value for each peak to describe the statistical significance of read intensity difference between the two samples being compared. M value = log2 (Read density in sample 1/Read density in sample 2). We therefore identified the significant upregulated or downregulated regions based on $P < 0.05$ and $|M| > 0$. To be visualized and comparable, we used signal track of each sample calculated by MACS2 and normalized by sequencing depth, and use Integrative Genomics Viewer (IGV) to set group tracks for each pair of the wild-type and $PA200^{-/-}$ sample. To determine the ChIP-seq average profile in genome and in up- or down-regulated genes, we used ngs.plot to calculate mean coverage of all regions, and then used R to visualize. The analyzed functional elements included TSS

(transcription start site) and gene body. For ChIP-seq with the anti-polymerase II antibody, two biological repeats are provided, while there is one sample for ChIP-seq with anti-H3K4me3 or anti-H3K56ac antibody.

Growth curve assay. Cell population doubling was determined as described [8]. Growth rates of primary MEFs were determined by microscopic measurement (OLYMPUS, CKX31) after staining by trypan blue according to the instructions.

Senescence-associated β-galactosidase (SA-β-gal) staining assay. Staining was performed using the Senescence Cells Histochemical Staining Kit (Sigma-Aldrich, #CS0030) according to manufacturer's instructions. Briefly, the cells were seeded at 3×10^5 cells per well in six-well plates for 24 h. The cells were then washed with 1×PBS and fixed with fixation buffer for 7 min at room temperature. The cells were then incubated overnight at 37 °C with the working solution containing 1 mg/ml X-gal in the kit, and senescence was identified as positive in the dark blue-staining cells observed. At least 100 cells were counted for each group in over three random fields to determine the percentage of SA-β-gal-positive cells.

8-arm radial maze. A group of animals were trained so that they would become habituated to the apparatus (Med Associates, ENV-256I) and food pellets for 3 days before each test. The mice were also treated with food limitation to reduce the body weight to 85%. In each training session, the animal was placed in a circular plastic wall on the platform in the middle of the 8-arm radial maze. Then, after 1 min, the ring was lifted, and the animal was allowed to move freely in the maze. The trial continued until the animal had either entered all 8 arms or until 10 min had elapsed. A small piece of popcorn (50 mg) was used as the bait. The performance of a given animal in each trial was assessed using three parameters: the number of correct choices in the initial 8 chosen

arms, the number of errors which was defined as choosing arms that had already been visited, and the time elapsed before the animal ate all 8 pellets.

Measurement of T cell surface marker expression. Spleens from mice were analyzed for the naive/memory CD4 and CD8 T cell subsets by flow cytometry using the following antibodies: CD4 (GK1.5; eBiosciences), CD44 (IM7; eBiosciences), CD62L (MEL-14; eBiosciences). Cells were acquired with a BD Biosciences FACS Aria IIIu instrument and analyzed using the FlowJo (Tree Star, Ashland, OR, USA) software package.

Fiber diameter measurements. Fiber diameter measurements were performed on cross sections of gastrocnemius muscles from the wild-type or PA200 deficient mice female mice at 3-month-old or 12-month-old. Gastrocnemius muscles were collected and processed for histology as described previously [9]. A total of 50 fibers were measured per muscle using Image J.

Determination of glomerular sclerosis. Formalin-fixed and paraffin-embedded kidney samples were stained using routine hematoxylin and eosin. Forty randomly selected glomeruli were scored for sclerosis. Glomeruli with > 50% sclerosis were determined to be sclerotic.

Statistical Analysis. All the images of immunoblotting were chosen blindingly and randomly and quantitated by image J. All values are expressed as the means ± SEM. from three independent biological replicates. One-way ANOVA was employed to compare values between multiple groups. Two-way repeated ANOVA was employed to compare multiple repeated measurements among groups.

Supplementary References

1. Li B, Dewey CN. RSEM: accurate transcript quantification from RNA-Seq data with or without a reference genome. BMC Bioinformatics. 2011; 12: 323.
2. Tarazona S, Furio-Tari P, Turra D, Pietro AD, Nueda MJ, Ferrer A, et al. Data quality aware analysis of differential expression in RNA-seq with NOISeq R/Bioc package. Nucleic Acids Res. 2015; 43: e140.
3. de Hoon MJ, Imoto S, Nolan J, Miyano S. Open source clustering software. Bioinformatics. 2004; 20: 1453-4.
4. Saldanha AJ. Java Treeview--extensible visualization of microarray data. Bioinformatics. 2004; 20: 3246-8.
5. Zhang S, Qin C, Cao G, Guo L, Feng C, Zhang W. Genome-wide analysis of DNA methylation profiles in a senescence-accelerated mouse prone 8 brain using whole-genome bisulfite sequencing. Bioinformatics. 2017; 33: 1591-5.
6. Young MD, Wakefield MJ, Smyth GK, Oshlack A. Gene ontology analysis for RNA-seq: accounting for selection bias. Genome Biol. 2010; 11: R14.
7. Mao X, Cai T, Olyarchuk JG, Wei L. Automated genome annotation and pathway identification using the KEGG Orthology (KO) as a controlled vocabulary. Bioinformatics. 2005; 21: 3787-93.
8. Liu GH, Qu J, Suzuki K, Nivet E, Li M, Montserrat N, et al. Progressive degeneration of human neural stem cells caused by pathogenic LRRK2. Nature. 2012; 491: 603-7.
9. Baker DJ, Perez-Terzic C, Jin F, Pitel KS, Niederlander NJ, Jeganathan K, et al. Opposing roles for p16Ink4a and p19Arf in senescence and ageing caused by BubR1 insufficiency. Nat Cell Biol. 2008; 10: 825-36.
